# Brain structure correlates of social information use: an exploratory machine learning approach

**DOI:** 10.3389/fnhum.2024.1383630

**Published:** 2024-07-02

**Authors:** Esra Cemre Su de Groot, Lieke Hofmans, Wouter van den Bos

**Affiliations:** ^1^Web Information Systems, Delft University of Technology, Delft, Netherlands; ^2^Developmental Psychology, University of Amsterdam, Amsterdam, Netherlands; ^3^Center for Adaptive Rationality, Max Planck Institute for Human Development, Berlin, Germany

**Keywords:** social information use, decision-making, pars triangularis, MRI, machine learning, brain structure

## Abstract

**Introduction:**

Individual differences in social learning impact many important decisions, from voting behavior to polarization. Prior research has found that there are consistent and stable individual differences in social information use. However, the underlying mechanisms of these individual differences are still poorly understood.

**Methods:**

We used two complementary exploratory machine learning approaches to identify brain volumes related to individual differences in social information use.

**Results and discussion:**

Using lasso regression and random forest regression we were able to capture linear and non-linear brain-behavior relationships. Consistent with previous studies, our results suggest there is a robust positive relationship between the volume of the left pars triangularis and social information use. Moreover, our results largely overlap with common social brain network regions, such as the medial prefrontal cortex, superior temporal sulcus, temporal parietal junction, and anterior cingulate cortex. Besides, our analyses also revealed several novel regions related to individual differences in social information use, such as the postcentral gyrus, the left caudal middle frontal gyrus, the left pallidum, and the entorhinal cortex. Together, these results provide novel insights into the neural mechanisms that underly individual differences in social learning and provide important new leads for future research.

## 1 Introduction

Most of our everyday decisions are influenced by social information. This information is gathered from, for instance, observing another individual spending money on a new car, advice from a peer for a nice holiday location, or a group of friends who all vote for the same political party. Although everybody relies on social information, some individuals prefer individual learning whereas others put more weight on the opinion of others. Social learning is driven by several contextual factors, including how certain individuals are about their decision (Morgan et al., 2012), task difficulty, and environmental change (Toelch et al., 2009). However, besides these contextual factors, prior studies have found that there are consistent individual differences in social learning strategies (Molleman et al., 2014) and social information use (Toelch et al., 2014b; Molleman et al., 2019), and these differences are consistent over long periods (e.g., 9 months; Molleman et al., 2019). This means that, within a given context, some individuals consistently use more social information than other individuals. Although individual differences in social information use affect many important social dynamics, such as the rate of polarization and cooperation in society, the underlying mechanisms of these individual differences in social learning are still poorly understood.

**Figure 1:**
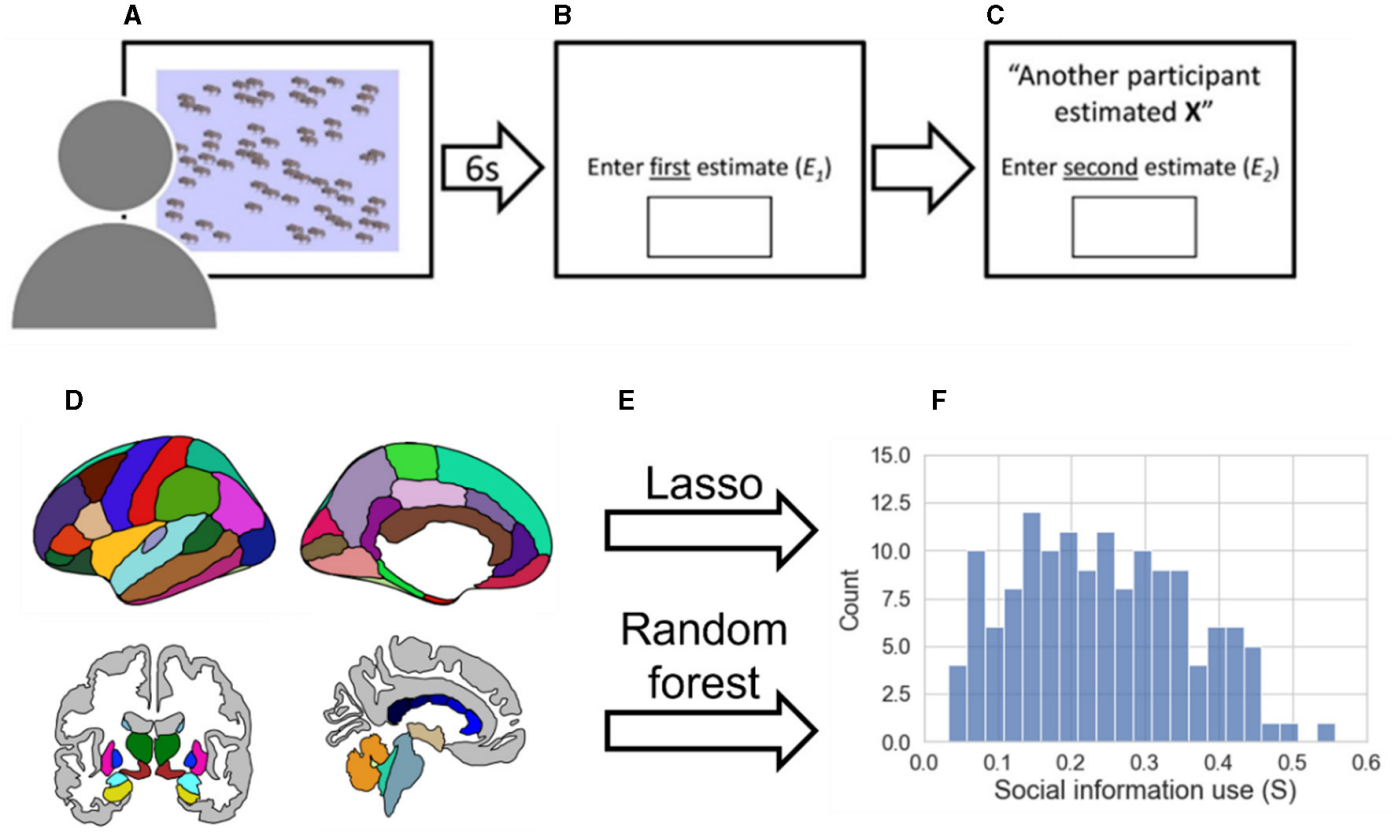
Methods pipeline. **(A)** During the BEAST (Molleman et al., 2019) participants viewed a number of animals on the screen for a period of 6 seconds. **(B)** Participants entered their first estimate (E_1_). **(C)** Participants are shown social information (X) and entered a second estimate (E_2_). **(D)** Gray matter volume parcellation of cortical and subcortical regions according to the Desikan/Killiany atlas (Desikan et al., 2006) are extracted with Freesurfer. **(E)** A lasso regression (linear) and random forest regression (non-linear) are trained using leave-one-out cross-validation to predict social information use. **(F)** Distribution of social information use excluding participants who never used social information (*n* = 141, mean = 0.2415, SD = 0.1164, median = 0.2366).

A more thorough understanding of which neural areas are involved in social information use would generate more insights into the cognitive mechanisms and computations that are recruited in this process (Olsson et al., 2020; Hofmans and van den Bos, 2022). Some previous functional neuroimaging studies have already looked into potential aspects of social information use. For example, areas that have been implicated in subjective confidence and the exploration of additional information, including the medial prefrontal cortex and the anterior cingulate cortex (De Martino et al., 2013; Lebreton et al., 2015), might be important in deciding if information provided by others would be of additional value. Furthermore, regions important for social cognition, including the temporo-parietal junction (Saxe and Kanwisher, 2003; Rushworth et al., 2013) and the medial prefrontal cortex (Amodio and Frith, 2006; Hampton et al., 2008; Mahmoodi et al., 2023) might be involved in deciding whose information to use, whereas the orbitofrontal and dorsolateral prefrontal regions have been found to play a role in the integration of information (Krawczyk, 2002; Bowman et al., 2012; Filimon et al., 2013; Pedersen et al., 2015; Nogueira et al., 2017). However, functional MRI measures have mediocre to poor test-retest reliability, rendering them suboptimal for researching individual differences (Elliott et al., 2020). In contrast, technological developments in the field of neuroimaging allow us to accurately and reliably quantify brain structure, with measures such as cortical volume showing high test-retest reliability (TRC = 0.88) (Iscan et al., 2015). Although the brain's structural-functional relationships are not yet fully understood, linking structure to behavior is an essential first step in constructing a fully interpretable neural phenotype (Llera et al., 2019). Importantly, we can also reliably measure individual differences in social information use. For example, the BEAST (see Figures 1A–C) has shown high test-retest reliability (r = 0.60) after nine months (Molleman et al., 2019). The availability of two reliable measures, brain structure and behavior, facilitates exploratory machine learning analyses to uncover previously hidden relationships between brain and behavior (Poldrack and Farah, 2015).

The current study aims to identify the structural neural phenotype that relates to individual differences in social information use. Given the long-term stability of individual differences in social learning, we hypothesize that different structural brain features, specifically regional variation in gray matter volume, are related to these consistent individual differences in social learning. Social information use is measured using a behavioral task in which participants can adjust their responses based on the responses given by others (Figures 1A–C). The measurement of social information use is then calculated by the shift from participants' first response toward the social information, which has been shown to be a robust measure of social learning (Molleman et al., 2019). By exploring the underlying structural volumes of these behavioral individual differences, we aim to partly reveal the brain-behavior relationship and contribute to building the neural phenotype of individual differences in social learning. The underlying structural brain regions that relate to individual differences can provide an interesting foundation for future research investigating how social learning relates to functional brain activity.

To identify cortical and sub-cortical volumes that show consistent variability with social information use, we trained two distinct types of exploratory machine learning models, Lasso regression and Random Forest regression, in combination with cross-validation (Figure 1). We believe an exploratory approach, in this case, is preferred to the typical confirmatory volume of interest (VOI) approach for several reasons. First, there are currently no neuroimaging studies that have focused on individual differences in social information use with sample sizes that would allow for reliable individual differences analyses. In addition, VOIs are often selected based on group-level results that are often not an accurate representation of the individual VOIs (Genon et al., 2017). Finally, the VOI approach is restricted and may result in missing out on yet unknown brain-behavior relationships. We used a cross-validated approach to prevent overfitting, and therefore increase reliability and generalizability. The Lasso and Random Forest regression are chosen to include a linear and non-linear model in our analysis. Adding the Random Forest regression allows us to capture possible interaction effects between different brain areas and other non-linear brain-behavior relationships, addressing the complexity of the brain. Therefore, our results are not limited to linear brain-behavior relationships.

## 2 Materials and methods

### 2.1 Participants

In this study, 188 students (mean age = 20.77, SD = 3.86, 50.5% female) from the University of Amsterdam participated. All participants were able to understand and communicate with a professional proficiency in English and were either Dutch or international students. Ethical approval was obtained from the Ethics Review Board of the Faculty of Social and Behavioral Sciences of the University of Amsterdam (ERB number: 2019-DP-10814). After giving informed consent, participants completed the computer task online and were subsequently asked to come to the Spinoza Centre for Neuroimaging for their MRI scan. Data cleaning (see Section 2.2) resulted in 141 participants (mean age = 20.18, SD = 1.83, 53.2% females) to model the data. Participants were paid €25 (this includes a monetary reward for two other computer tasks and questionnaires that are not part of the current study) plus up to €1 bonus.

### 2.2 Social information use task

Social information use was measured using the Berlin Estimate AdjuStment Task (the BEAST; Figures 1A–C) (Molleman et al., 2019). Participants performed the task online, which took ~5 min to finish. To increase participants' motivation to perform well on the task, participants received a performance-based monetary bonus of up to €1, based on a random trial (first or second estimate).

During the BEAST, participants saw a number of animals for 6 s, after which they entered their first estimate (E_1_) of the number of animals. Afterward, participants saw the estimate given by a previous participant, the social information (X). The targeted value of the estimate of the other participant (X) that was shown was calculated as follows:


$$\begin{array}{l} X=E_{1}\times \left(1\;\pm \text{ Δ}\right) \end{array}$$

The value of Δ was kept at 0.20, such that the social information was always 20% toward the true number of animals. Thus, the value of Δ was added to 1 if the first estimate (E_1_) was lower than the true number of animals and subtracted from 1 if E_1_ was higher than the true number of animals. If participants accurately estimated the exact true number of animals, the direction of the social information was chosen randomly. Note that it was not always possible to show social information that deviated exactly 20% from the first estimate, because the social information stemmed from real previous participants (from prior experiments using the BEAST). In these cases, the estimate of another participant that was closest to the targeted social information was shown, which deviated mostly one or two animals away from the 20% point. The absolute error of the first estimate was calculated by taking the absolute difference between the first estimate and the real number of animals. After viewing the social information, participants gave a second estimate (E_2_). Social information use per trial was calculated using the following formula:


$$\begin{array}{l} s_{trial}=\;\frac{E_{2}-E_{1}}{X-\;E_{1}} \end{array}$$

All participants completed 5 trials. Social information use (S) measured by the BEAST shows low variability between trials and maintains consistency over months (Molleman et al., 2019). Trials with a value for social information use (s_trial_) smaller than 0 or bigger than 1 were excluded (Molleman et al., 2019) because this type of behavior does not capture weighing their estimate with the presented social information. On top of that, trials with a reaction time of 20 s or longer for entering the first estimate were excluded, because in these trials, accuracy was often either very high or very low. A very high accuracy raises the possibility that those participants might have taken screenshots and counted the number of animals, whereas a very low accuracy might indicate that participants with such a long reaction time were distracted during that trial. These exclusion criteria resulted in some participants having fewer than 5 trials. Therefore, we set a minimum of 3 trials for participants to be included (Gradassi et al., 2023). This resulted in the exclusion of 17 participants. The mean social information use (S) and the mean absolute error per participant were then calculated by taking the mean of all trials left. After removing age outliers (2 standard deviations from the mean), 159 participants were left. Of those 159 participants, 18 participants did not use any social information in any of the trials. This caused the data to be not normally distributed (mean S = 0.2141, SD = 0.1340, median = 0.2133), differing from the distribution of S in the research of Molleman et al. (2019). Therefore, the data of these 18 participants were considered to be unreliable and were excluded from the main analysis (an analysis including these participants can be found in the Supplementary material). This resulted in a final number of 141 participants (mean S = 0.2415, SD = 0.1164, median = 0.2366, Figure 1F).

### 2.3 MRI data acquisition

Structural imaging data were collected using a 3 Tesla MRI scanner (Philips Achieva DS, 32 channel head coil) at the Spinoza Centre for Neuroimaging. The scan included two high-resolution T1-weighted anatomical scans (voxel size = 0.70 × 0.81 × 0.70 mm, FOV = 256 × 256 × 180 mm, matrix size = 368 × 318 × 257 slices, TR = 11 ms, TE = 5.2 ms, flip angle = 8°, parallel acquisition technique = SENSE), which were averaged.

### 2.4 MRI data preprocessing

The MRI data were automatically pre-processed using *fMRIPrep* 1.5.4 (Esteban et al., 2018a,b; RRID:SCR_016216), which is based on Nipype 1.3.1 (Gorgolewski et al., 2011, 2018; RRID:SCR_002502) in combination with Freesurfer (http://surfer.nmr.mgh.harvard.edu/). A more detailed description of the preprocessing with *fMRIPrep* can be found in the Supplementary material.

### 2.5 Gray matter volume extraction

Gray matter volumes of all brain areas of both hemispheres (cortical and subcortical) corresponding to the Desikan/Killiany (DK) atlas (Desikan et al., 2006) and Freesurfer's Aseg atlas were extracted (Fischl et al., 2002) using Freesurfer. Subsequently, the gray matter volumes of all brain areas (89 areas) were scaled to account for brain size using the *SupraTentorialVolNotVent* parameter, which includes gray matter and white matter volumes of the brain (excluding cerebellum, brain stem, ventricles, CSF, and choroid plexus). Ventricles were subtracted from the total brain volume as the size of the ventricles influences white and gray matter volume. Moreover, the cerebellum was not taken into account because, during the scanning procedure, the cerebellum was often cut off the scan when trying to fit the entire brain into the field of view. Brain areas that occur in both hemispheres were averaged into one brain area if the volume of both areas of the hemispheres had a Pearson's correlation of 0.7 or higher. This was done to prevent collinearity between the brain regions, which is important for the interpretability of the regression model. Moreover, it is likely to assume that bilateral brain regions that highly correlate with each other share the same predictability toward social information use. Because of this criterium, 6 brain areas were reduced, resulting in a final number of 83 brain areas.

For our main analysis, we chose to use an atlas-based ROI approach to increase statistical power and reduce the multiple comparison problem that is associated with voxel or vertex-wise approaches, and for the possibility of including subcortical regions (Backhausen et al., 2022). Collinearity can decrease the reliability of the relative importance of certain features (Dormann et al., 2013), which we are particularly interested in. Furthermore, due to the sheer number of vertices or voxels, such approaches are currently not feasible for the machine learning techniques used for feature extraction (see Supplementary material for vertex-based single-order correlations^1^). For the same reason, we chose to use the DK atlas instead of other atlases with more fine-grained parcellation, such as the Destrieux atlas (Destrieux et al., 2010) or the Glasser atlas (Glasser et al., 2016).

### 2.6 Feature extraction

We modeled a Lasso regression (linear) and Random Forest (RF) regression (non-linear) to explore which brain areas play a role in the neural processing of social information use. To identify the brain regions related to social information use, the coefficients from the Lasso regression and feature permutation importance from the RF regression were used. The volumes of 83 regions, as well as the control variables age and sex, were included as features (predictors). Lasso regression was chosen based on its characteristics to discard features (reduce them to zero) that do not contribute to the model prediction. We initially ran a more computationally costly elastic-net regression, which combines Lasso with Ridge regression (which sets features to near zero rather than exactly zero). However, because the elastic-net regression kept leaning toward a full Lasso regression, we ultimately decided to perform Lasso regression. The RF regression was chosen based on its robustness and ability to capture non-linear relationships (Breiman, 2001), while Lasso regression is restricted to identifying linear relationships. The RF regression uses an ensemble of decision trees and different bootstrapped samples of the data and a different set of features for each decision tree. All the trees of the forest produce a prediction, which is averaged into one final prediction.

The Lasso and RF models were trained using scikit-learn version 0.24.2 (Pedregosa et al., 2011) in Python. Before training the model, all features were scaled between 0 and 1 to make them comparable. We applied a Leave One Out Cross-Validation (LOOCV) outer loop, where one participant in each loop was used to evaluate the model, which was trained on the remaining participants (n – 1). This resulted in 1 model per participant (n = 141). We preferred this method over lower-fold cross-validation (e.g., 5-fold or 10-fold) because the current dataset was relatively small compared to regular datasets used within machine learning algorithms, which often consist of at least 10,000 instances. Using LOOCV, the number of data points to fit the model was increased compared to lower-fold cross-validation, and therefore the bias of the model and the chance of overfitting were reduced. Moreover, each subject in the dataset contributes to the estimation of the model performance. Of note, because each training set is so similar (differing n – 2 instances), the models resulting from LOOCV are mutually dependent which could be a risk for overfitting.

For the Lasso regression, we additionally applied a 5-fold inner loop for a grid search to finetune the hyperparameter alpha that controls the L1 regularization, which regulates how readily coefficients of features are set to zero. Values of alpha ranging between 0.001 and 0.999 with a step size of 0.001 were searched to find the optimum value of alpha. For the RF regression, we increased the number of decision trees to 1000 to increase reliability and robustness but kept other hyperparameters at default to reduce computational costs. RF regression produces a measure of importance per feature, meaning how important they are in explaining the dependent variable, here social information use. As the standard measure of feature importance, based on feature impurity, is not always reliable (Strobl et al., 2007), we assessed another metric called permutation importance (Breiman, 2001; Altmann et al., 2010). This was computed using the difference in model performance, mean squared error (MSE), between including the actual vs. permuted (random) values for a feature. Permutation importance was averaged over five permutations to account for random values being meaningful by chance. MSE should increase when permuting important features, resulting in high permutation importance. The coefficients (Lasso), permutation importance (RF), and model performance (MSE) of each model were stored, resulting in a distribution of these measures resulting from each loop of the LOOCV.

### 2.7 Comparison with baseline

A baseline feature “RANDOM” was computed. The values of this baseline feature consisted of random numbers between 0 and 1 and should thus not be relevant for predicting social information use. The relevance of other features (brain areas) can therefore be derived based on a comparison with the baseline feature. To make sure this randomized feature was not accidentally important by chance, its values were newly computed for each loop within the LOOCV. The distributions of the Lasso coefficients and RF permutation importance are visually compared with the baseline feature. We did not test for significant differences as the observed coefficients and importance scores are not independent observations, making statistical testing of relative importance unreliable. Furthermore, due to the exploratory nature of our analysis, no confirmatory conclusions can be drawn upon statistical significance. Therefore, we believe a visual inspection of the differences of the coefficients and importance scores of the brain regions with the baseline feature is an appropriate method to derive meaningful leads for future confirmatory research.

Moreover, although it is not expected that machine learning models can precisely predict behavior solely on brain volumetric measures, and the machine learning models in this study are used rather exploratorily, it is still interesting to put the model performance in perspective. Therefore, we additionally created a baseline model that solely included the average social information use from the train data to predict the evaluation data. This way, we could compare the MSE for the simple, but not meaningless, baseline model with the more complex Lasso and RF models.

## 3 Results

### 3.1 Lasso regression

**Figure 2:**
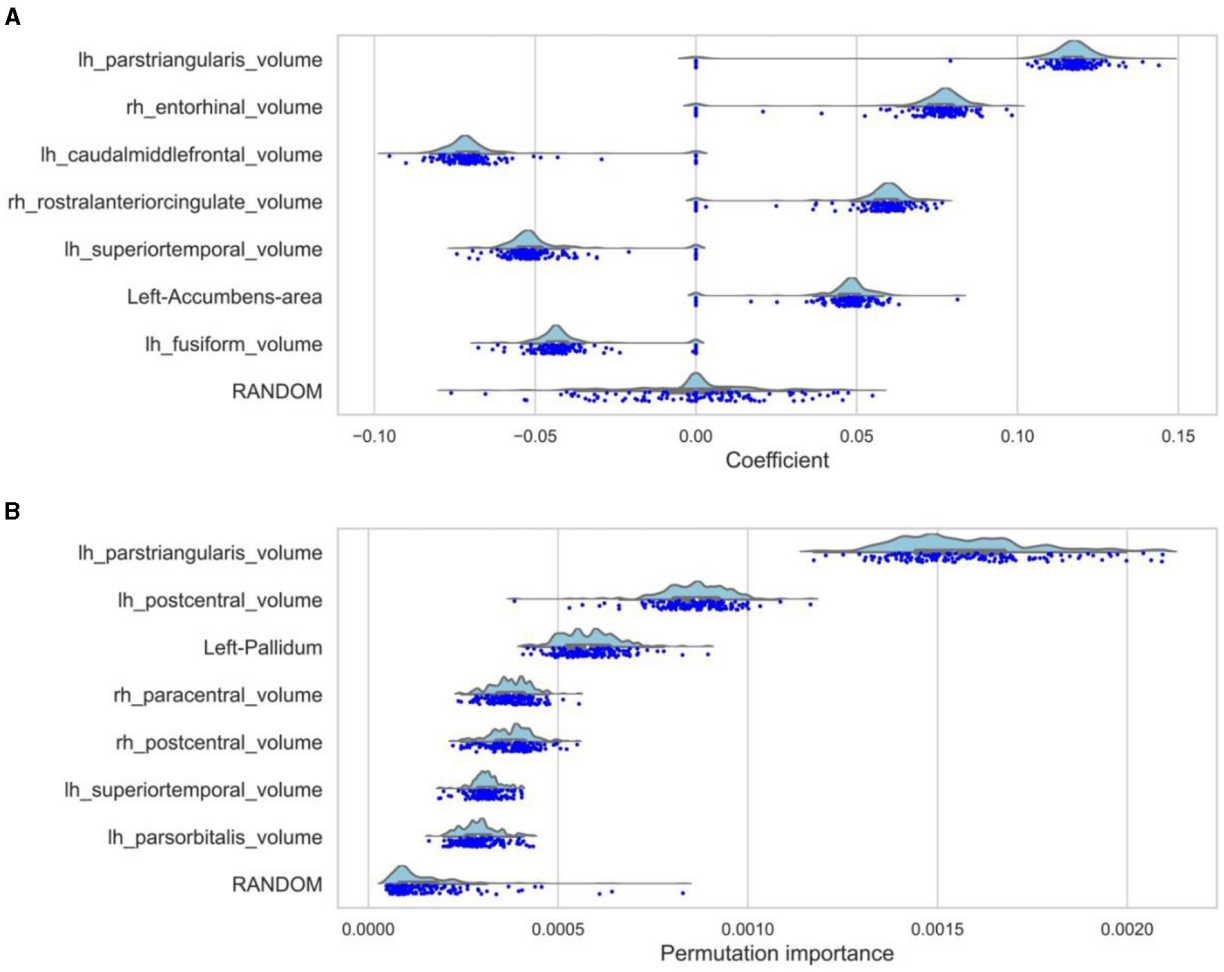
Top 7 feature importances and coefficients distributions. **(A)** The distributions of the seven biggest coefficients together with the baseline feature (RANDOM) of the winning lasso model. This model only contains the non-zero features from the first model. Therefore, the zero-peaks are much smaller because there is less noise from unimportant features and collinearities. The dots represent the coefficients based on the individual loocv runs. **(B)** The distributions of the importances resulting from the loocv of the random forest regression are shown for the seven features with the highest mean importance together with the baseline feature (RANDOM).

**Figure 3:**
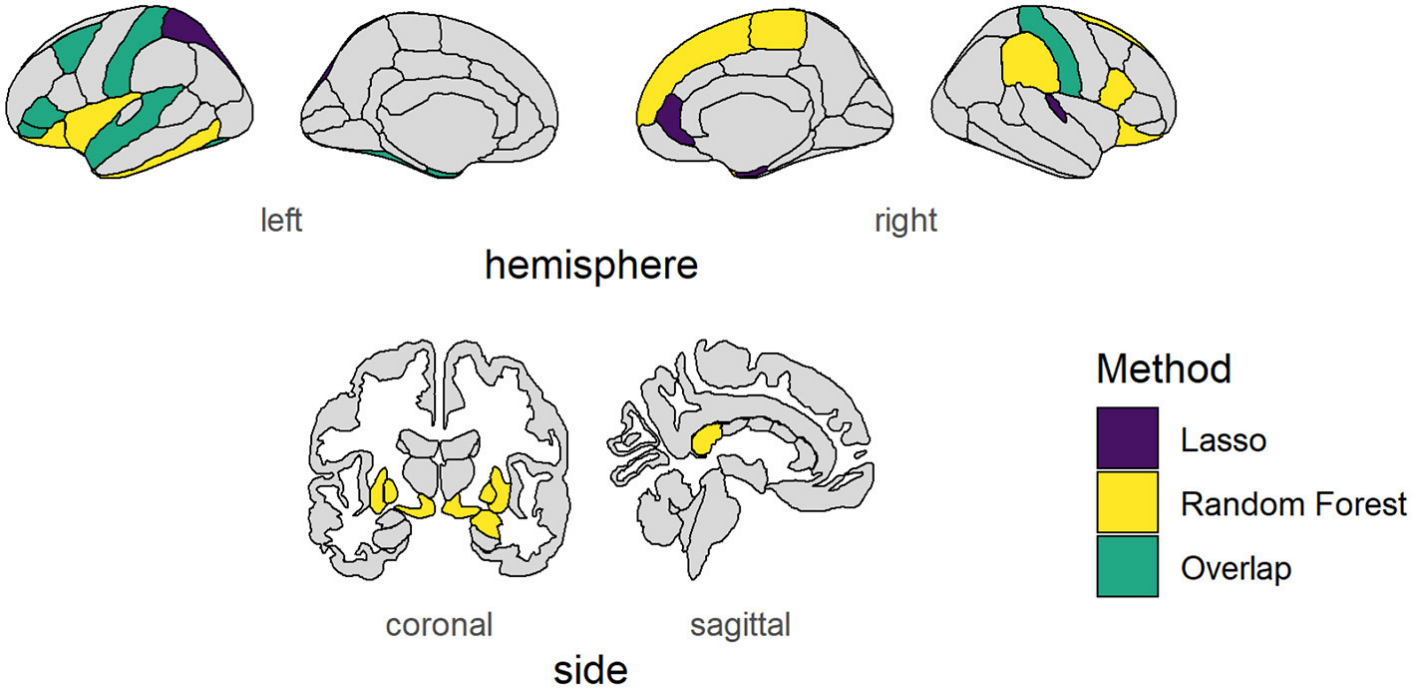
Brain areas vary with social information use. Cortical and subcortical brain areas of which their volumes showed consistent variability with our behavioral measure of social information use as found using Lasso regression (purple), Random Forest regression (yellow), or both (green). NB: The left nucleus accumbens showed consistent variability according to both measures, but falls outside the depicted brain slices and is therefore not shown.

Using Lasso regression, 14 brain regions, the baseline feature RANDOM, and sex had non-zero mean coefficients (Supplementary Figure S1A, Supplementary Table S1). The lasso regression showed peaks at zero because the lasso regression is sensitive to interference between brain regions. Therefore, the lasso regression was rerun with only the features that had a non-zero mean coefficient (referred to as the “winning model”). This reduced the noise of unimportant features and reduced possible interference between brain regions. The distributions of the seven biggest mean coefficients as a result of the winning lasso model are shown in Figure 2A, a figure and table containing coefficients of all features can be found in the Supplementary Figure S1B, Supplementary Table S2. The brain areas that had a non-zero mean coefficient are visualized in Figure 3. The gray matter volume of the left pars triangularis had the highest absolute mean coefficient (mean β = 0.1109, SD = 0.0280), followed by the gray matter volume of the right entorhinal cortex (mean β = 0.0720, SD = 0.0196), and the left caudal middle frontal gyrus (mean β = −0.0672, SD = 0.0181). The baseline feature had a mean coefficient of −0.0006 (SD = 0.0216).

### 3.2 Random forest regression

RF regression revealed 24 brain regions with a higher mean permutation importance (*i*) than the RANDOM baseline feature (Supplementary Figure S2A, Supplementary Table S3). These brain areas are visualized in Figure 3. The distribution of the top seven brain areas with the highest permutation importance is shown in Figure 2B. The brain area with the highest mean permutation importance was again the left pars triangularis (mean *i* = 0.0016, SD = 0.0002), followed by the left postcentral gyrus (mean *i* = 0.0009, SD = 0.0001) and the left pallidum (mean *i* = 0.0006, SD = 0.0001). The baseline feature had a mean permutation importance of 0.0002 (SD = 0.0001). In line with the robust character of the RF regression, running the model again with only features with a higher mean permutation importance than the baseline feature (the winning model) did not change the order of the feature importance, but only increased the overall importance value (Supplementary Figure S2B, Supplementary Table S4).

### 3.3 Model performance

The Lasso (MSE = 0.0143, SE = 0.0014) and RF model (MSE = 0.0141, SE = 0.0014) resulted in lower model performance than the simple baseline model (MSE = 0.0136, SE = 0.0013). We argued that this might be due to a high level of noise resulting from the multitude of included features. Indeed, when looking at the winning Lasso model which only included those features with non-zero mean coefficients, model performance is increased relative to the baseline model (MSE = 0.0128, SE = 0.0013). Similarly, the winning RF model, which only included those features with a higher permutation importance than the baseline feature, performed better (MSE = 0.0123, SE = 0.0012). Supplementary Figure S5 shows the distribution of the MSE of all the models. Thus, both the Lasso and RF regression do not outperform the baseline model when including all features. However, when reducing noise by removing non-important features, both models slightly outperform the baseline model.

### 3.4 *Post-hoc* correlations with social information use

**Figure 4:**
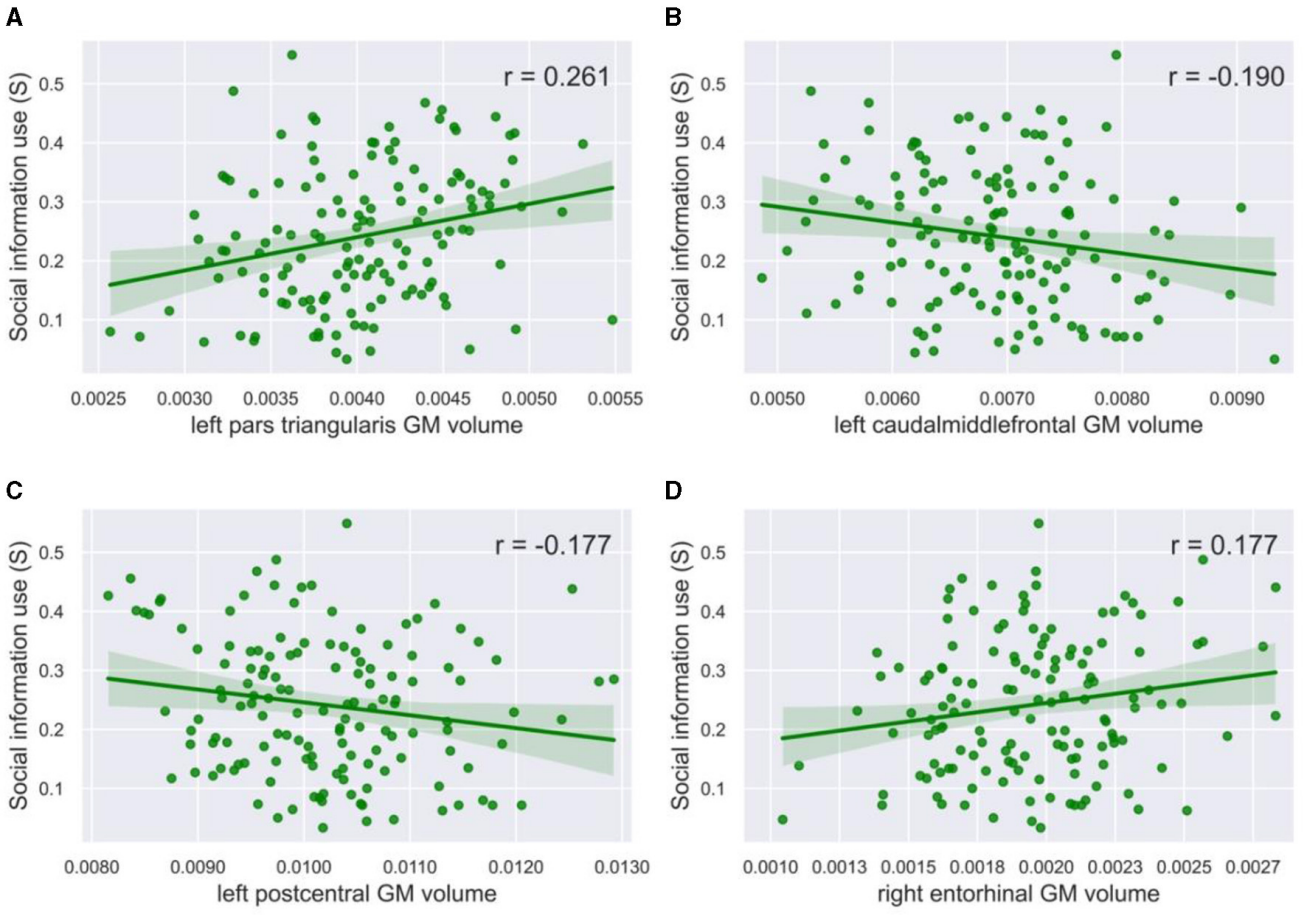
Correlation plots with social information use. The gray matter volume of **(A)** the left pars triangularis (r = 0.261, p = 0.002, α = 0.05) and **(D)** the right entorhinal cortex (r = 0.177, p = 0.032, α = 0.05) showed a significant positive correlation with social information use. The gray matter volume of **(B)** the left caudal middle frontal gyrus (r = −0.190, p = 0.024, α = 0.05) and **(C)** the left postcentral gyrus (r = −0.177, *p* = 0.036, α = 0.05) showed a significant negative correlation with social information use. The green line in the plots shows the slope of the correlation.

To gain a better insight into the results from the Lasso and RF models, especially because the relationships found using RF regression are not necessarily linear, we visualized the relationship between gray matter volumes and social information use (Figure 4). Based on visual inspection, we did not find any clear non-linear relationships. When investigating linear relationships, we found a significant positive Pearson's correlation for the volume of the left pars triangularis (r = 0.261, *p* = 0.002, α = 0.05, Figure 4A), and a negative correlation for the left caudal middle frontal gyrus (r = −0.190, *p* = 0.024, α = 0.05, Figure 4B), and the left (r = −0.177, *p* = 0.036, α = 0.05, Figure 4C) and right (r = −0.180, *p* = 0.032, α = 0.05, not visualized) postcentral gyrus with social information use. A positive correlation was also found for the right (r = 0.177, *p* = 0.035, α = 0.05, Figure 4D) and the left (r = 0.173, *p* = 0.040, not visualized) entorhinal cortex. The correlation between social information use and the volume of the left pallidum was not significant (r = 0.164, *p* = 0.052, α = 0.05).

### 3.5 *Post-hoc* correlations with task performance

**Figure 5:**
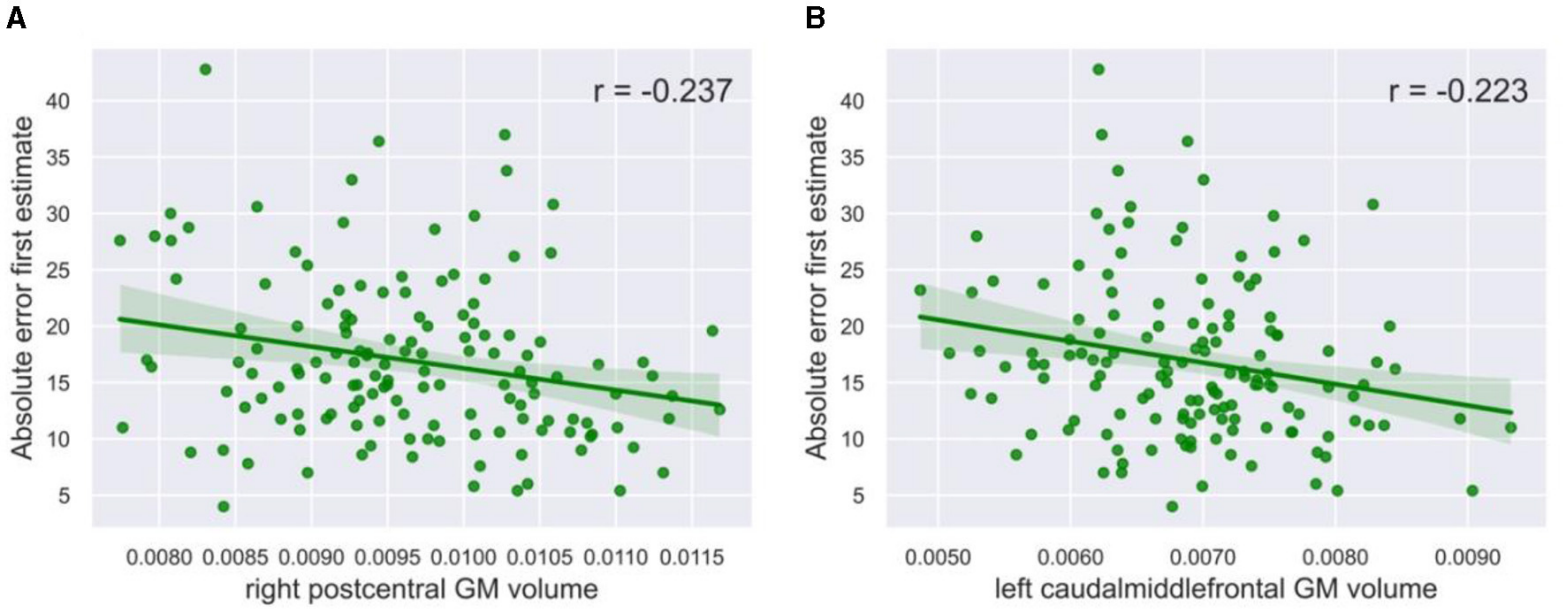
Correlation plots with task performance. The gray matter volume of **(A)** the right postcentral gyrus (r = −0.237, *p* = 0.005, α = 0.05) and **(B)** the left caudal middle frontal gyrus (r = −0.223, *p* = 0.008, α = 0.05) showed a significant negative correlation with social information use. The green line in the plots shows the slope of the correlation.

While the postcentral gyrus is, to our knowledge, not necessarily related to social processes, it has been implicated in visual processing (Tomasi et al., 2007; Wang et al., 2008). This raises the speculation that the postcentral volume, and possibly also other brain regions, might be related to performance on our visual task, rather than the process of weighing social information toward individual information. The volume of the right postcentral gyrus indeed showed a significant negative correlation with the absolute error of the first estimate (r = −0.237, *p* = 0.005, α = 0.05, Figure 5A). Likewise, the volume of the caudal middle frontal gyrus negatively correlated with the absolute error of the first estimate (r = −0.223, *p* = 0.008, α = 0.05, Figure 5B). A lower absolute error might in turn have led to less social information use, although the direct correlation between absolute error and social information use was not significant (r = 0.157, *p* = 0.062, α = 0.05). The left pars triangularis (r = 0.030, *p* = 0.726, α = 0.05), the left postcentral gyrus (r = −0.101, *p* = 0.232, α = 0.05), the left pallidum (r = 0.042, *p* = 0.624, α = 0.05), and the right entorhinal cortex (r = 0.135, *p* = 0.111, α = 0.05) did not correlate with absolute error, suggesting that they are uniquely related to social learning.

## 4 Discussion

We aimed to find cortical and sub-cortical brain areas that show consistent variability with individual differences in social information use. Two complementary machine learning approaches—Lasso regression and Random Forest (RF) regression—were trained to predict social information use at the individual level using 83 gray matter volumes of cortical and subcortical brain areas of the DK atlas, age, and sex as predictors. Based on these models, a small set of brain regions turned out to be associated with social information use, including the volumes of the left pars triangularis, the left caudal middle frontal gyrus, the left postcentral gyrus, the right entorhinal cortex, and the left pallidum showing in the top three of our models.

Interestingly, based on both models, the left pars triangularis robustly appeared to be an important brain region: the gray matter volume of the left pars triangularis was positively associated with social information use. The left pars triangularis is part of the left inferior frontal gyrus (LIFG), together with the pars opercularis and pars orbitalis. In line with our results, prior functional neuroimaging research on social learning has shown that neural activation in the LIFG and the left pars triangularis in particular, is related to the efficient integration of social and individual information during a perceptual decision-making task under uncertainty (Toelch et al., 2014a). They propose an inhibitory role for the LIFG toward using *individual* information at moments when using *social* information is more valuable. Another study found that greater activity in the LIFG is related to a greater shift in response bias during a decision-making task (Reckless et al., 2014), thereby again being related to flexibility in decision-making. Moreover, the IFG also seems to play a role in perceptual social information processing, more specifically in perceptual emotion recognition (Keuken et al., 2011) and processing information about the self vs. the other (Kircher et al., 2000). Together, these results provide corroborating evidence for the left pars triangularis to play a role in valuing social information over individual information during our experimental task.

Next, in contrast with the pars triangularis, the left postcentral gyrus and the left caudal middle frontal gyrus both showed a negative relationship with social information use. Prior research has shown that neural activation in the postcentral gyrus is related to visual processes in the brain (Wang et al., 2008), including visual attention (Tomasi et al., 2007) and mathematical approximation (Dehaene et al., 1999). More specifically, prior research has found that less gray matter volume of the postcentral gyrus relates to a better perception of numerosity in a decision-making task (Yuan et al., 2023). A sense of numerosity may play an important role when performing the BEAST, as participants had to estimate the number of presented animals. Interestingly, we have observed a negative correlation between social information use and the gray matter volume of the postcentral gyrus, which would indicate that a greater sense of numerosity does not necessarily relate to lower social information use. Furthermore, activation in the caudal middle frontal gyrus seems to be related to contextual control during visual behavioral tasks (Nee and D'Esposito, 2016) and the ability to use higher cognitive function when selecting visual targets in a goal-directed way, using internal knowledge to implement a beneficial visual attention strategy (Germann and Petrides, 2020). Therefore, we speculated that the volume of the postcentral gyrus and the caudal middle frontal gyrus may be related to attention and task performance, rather than directly to the social aspect of social information use. Participants with high estimation accuracy—and awareness of their accuracy—might use less social information because they are more certain about their estimate (Morgan et al., 2012). Our *post hoc* analysis of task performance indeed showed that the right postcentral gray matter and the left caudal middle frontal gyrus volumes negatively correlate with the absolute error of the first estimate. However, future studies are needed to further investigate this relationship with a task that is more directly designed to measure performance accuracy and confidence [e.g., a moving-dot task with different confidence levels (Moussaïd et al., 2017)].

Our study also identified a positive relationship between the entorhinal cortex and social information use. The entorhinal cortex is usually related to the memory system, and more specifically to spatial representation (Fyhn et al., 2004). Interestingly, prior work in rodents has also found that the entorhinal cortex plays a role in the processing of social information for social cognition (Leung et al., 2018; Lopez-Rojas et al., 2022). Their work showed that the lateral entorhinal cortex provides direct input that is necessary for social cognition to the CA2 hippocampal region. Together with our findings, it seems like the entorhinal cortex plays a role in the processing of social information in humans. Further confirmatory research is necessary to further investigate if and how the entorhinal cortex might be related to processing social information in humans.

Finally, we remain agnostic as to the functional involvement of the left pallidum. Even though the basal ganglia, including the pallidum, have often been implicated in motivation, learning, and action-selection (Collins and Frank, 2014; Shipp, 2017), we are unaware of any robust direct relationships with social information use. More research, perhaps involving functional activity, is necessary to further understand the different roles of each brain area within social information use processes. Interestingly, the left pallidum was found to be an important predictor for social information use using RF regression, but not Lasso regression. An explanation could be that the relationship between the left pallidum and social information use is non-linear, as indicated by their non-significant linear correlation, or has more complex interactions with other brain areas and is therefore not detectable by the Lasso regression. Without using the RF regression, the left pallidum would have been missed, highlighting the added value of using RF models in exploring brain areas related to social information use. Due to the complexity of an RF model, it is difficult to interpret how the left pallidum relates to individual differences in social information use.

Prior studies in humans and rodents, researching social learning and cognition in the brain, have found a set of brain areas that overlap with our results. These areas include the medial prefrontal cortex (mPFC) (Amodio and Frith, 2006; Apps and Ramnani, 2017; Olsson et al., 2020; Zhang and Gläscher, 2020), the temporal parietal junction (TPJ) (Carter et al., 2012; Olsson et al., 2020; Zhang and Gläscher, 2020), the superior temporal sulcus (STS) (Amodio and Frith, 2006; Olsson et al., 2020), and the anterior cingulate cortex (ACC) (Amodio and Frith, 2006; Chang and Sanfey, 2013; Apps et al., 2016; Olsson et al., 2020; Zhang and Gläscher, 2020). Looking at our results, the left superior temporal cortex appears in the top seven of the RF and Lasso model. The right rostral anterior cingulate cortex occurs in the top seven of the lasso regression, overlapping with the ACC and most likely the mPFC. The right supramarginal gyrus shows up in the results of the RF regression at place 15 and partly overlaps with the TPJ.

While the social brain network regions largely occur in our results, the coefficients and importances of these brain areas are not that strong and are outperformed by (some novel) areas, such as the left pars triangularis, the left caudal middle frontal gyrus, the left postcentral gyrus, the right entorhinal cortex, and the left pallidum. One explanation could be that social brain network regions, such as mPFC, STS, TPJ, and ACC, are mainly involved in the process of tracking the context around social information, such as the mental states of other individuals, and using this information to decide whether to use the social information or not. During the BEAST, participants did not have any contextual information about the source of the social information. As a result, the current experiment could be merely a measurement of the weight of social information compared to individual information. This could explain why the current study finds different more important brain areas related to the processes of social information use, corresponding with studies that focus on the consideration of individual vs. social information (Toelch et al., 2014a).

Despite the goal of the current study not being to generate the most accurate predictive model but rather to explore different brain regions, we do want to highlight the importance of using a simple baseline model to put model performance in perspective. The current study uses a simple mean score as a baseline model to assess any added predictive value of more complex machine learning models. The mean model assumes that all participants have the same underlying social information use; all variance in social information use is based on measurement error. However, this is not consistent with the high test-retest reliability of social information use. Still, for purposes of estimating the relative contribution of neural data for the prediction of behavior, it can be a useful benchmark. Often, a baseline model is not used in neuroscientific research. Without this comparison, we have no idea to what extent the reported brain regions contribute meaningfully to explaining variance in behavior. Looking at the results of the current study, we see that the model performance of both of our complex models (lasso MSE = 0.0143, SE = 0.0014; RF MSE = 0.0141, SE = 0.0014) did not show an improvement compared to the predictive value of the mean social information use in the training set (our baseline model; MSE = 0.0136, SE = 0.0013). However, when reducing the noise of the model by keeping only those features with non-zero coefficients (Lasso) or by removing features that were less meaningful than a random feature (RF), the model performance of our complex models slightly improved with respect to the baseline model (Lasso MSE = 0.0128, SE = 0.0013; RF MSE = 0.0123, SE = 0.0012).

### 4.1 Limitations and future directions

As the brain volumes related to social information use found in our results are based on an exploratory approach, further confirmatory research is necessary to generalize the results and investigate the precise underlying brain-behavior relationships. First, it is not certain that the found structural volumetric neural individual differences related to social information use also translate to functional differences in the brain. The precise relation between regional volume and brain function is not fully understood, which makes it difficult to interpret how our findings relate to brain function. We have used structural differences in gray matter volume to identify ROIs related to consistent behavioral individual differences in social information use. While static structural volume might be useful to capture the consistent individual differences in social information use due to its replicability (Iscan et al., 2015), static measures might not be suitable to capture individuals' flexibility in social information use when it comes to specific situations. Together, further research using functional measures is necessary to investigate how the identified gray matter volume ROIs relate to brain function and the flexible nature of social information use.

As we have used the DK atlas to parcellate cortical brain regions, we are unaware of how the results would look when using a more fine-grained atlas. As mentioned in Section 2.5, we chose the DK atlas to increase statistical power and reduce problems related to (multi)collinearity (Dormann et al., 2013). On the other hand, an atlas with a more fine-grained parcellation, such as the Destrieux atlas, or the Glasser atlas, could have offered more precise insights into the specific brain areas related to social information use. However, an increase in parcellations might also increase the chances of collinearity between different brain areas and social information use. Furthermore, different types of parcellations could relate differently to social information use. Therefore, using a different atlas might result in different insights.

While our study has shown the added value of using a more complex model in identifying regions of interest, the interpretation of such black box models is often more difficult. Random forest models use a combination of multiple randomized decision trees (Biau and Scornet, 2016), which makes it possible to capture complex non-linear relationships between features, such as interactions between multiple features. Without a priori knowledge of how this complexity between multiple features might look, it is difficult to find the precise relationships between features within such a model, as the interaction structure can be complex (Boulesteix et al., 2012). The brain areas identified by our random forest model, and not by the lasso model, might involve more complex non-linear relationships with social information use. However, based on our results, we are not able to unravel the nature of these complex relationships.

Furthermore, while several tasks to measure information use exist, we have focused only on one task, the BEAST. To validate the generalizability of our results, it would be interesting to further investigate whether structural brain correlates with other social information use tasks [whose measures previously also showed a correlation with the BEAST (Molleman et al., 2019)], such as the moving dots task (Moussaïd et al., 2017) or bandit task (McElreath et al., 2005). Using multiple tasks also allows us to further investigate more precisely to what type of behavior the found brain regions relate. For example, it would be interesting to test whether a social information use task that involves an understanding of the context of the presented social information causes the social brain regions (the mPFC, STS, TPJ, and ACC) to become stronger predictors for individual social information use compared to the current results.

### 4.2 Conclusions

In sum, we find robust results that the gray matter volume of the left pars triangularis is associated with individual differences in social information use. While less robust than the left pars triangularis, there are novel brain areas found that are related to social information use, together with some common social brain regions. Further confirmatory research is necessary to investigate more precisely how these brain regions are related to social information use and validate the generalizability of the current results. To accomplish this, future research should include a wider diversity of behavioral measurements and measurements of connectivity and functional activity within the brain. Furthermore, the exploratory machine learning approach used in this study to link brain volumes with behavior can be used as a pipeline in future research to explore associations between brain structure and behavior and mark regions of interest.

## Data availability statement

The datasets presented in this study can be found in online repositories. The names of the repository/repositories and accession number(s) can be found below: https://github.com/sudegroot/200SCANS.

## Ethics statement

The studies involving humans were approved by the Ethics Review Board of the Faculty of Social and Behavioral Sciences of the University of Amsterdam (ERB number: 2019-DP-10814). The studies were conducted in accordance with the local legislation and institutional requirements. The participants provided their written informed consent to participate in this study.

## Author contributions

EG: Writing – original draft, Writing – review & editing, Conceptualization, Data curation, Formal analysis, Investigation, Methodology, Project administration, Resources, Software, Validation, Visualization. LH: Writing – original draft, Writing – review & editing, Formal analysis, Methodology, Supervision, Validation, Visualization. WB: Writing – original draft, Writing – review & editing, Conceptualization, Formal analysis, Funding acquisition, Investigation, Methodology, Project administration, Resources, Supervision, Validation, Visualization.

## Footnotes

^1^See “Vertex-wise analysis of the relationship between social information use and cortical brain volume” in our Supplementary material, including Supplementary Figure S6.

## References

[B1] Altmann A. Toloşi L. Sander O. Lengauer T. (2010). Permutation importance: a corrected feature importance measure. Bioinformatics 26, 1340–1347. 10.1093/bioinformatics/btq134 20385727

[B2] Amodio D. M. Frith C. D. (2006). Meeting of minds: the medial frontal cortex and social cognition. Nat. Rev. Neurosci. 7, 268–277. 10.1038/nrn1884 16552413

[B3] Apps M. Ramnani N. (2017). Contributions of the medial prefrontal cortex to social influence in economic decision-making. Cereb. Cortex 27, 4635–4648. 10.1093/cercor/bhx183 28922858

[B4] Apps M. A. Rushworth M. F. Chang S. W. (2016). The anterior cingulate gyrus and social cognition: tracking the motivation of others. Neuron 90, 692–707. 10.1016/j.neuron.2016.04.018 27196973 PMC4885021

[B5] Backhausen L. L. Herting M. M. Tamnes C. K. Vetter N. C. (2022). Best practices in structural neuroimaging of neurodevelopmental disorders. Neuropsychol. Rev. 32, 400–418. 10.1007/s11065-021-09496-2 33893904 PMC9090677

[B6] Biau G. Scornet E. (2016). A random forest guided tour. Test 25, 197–227. 10.1007/s11749-016-0481-7

[B7] Boulesteix A. L. Janitza S. Kruppa J. König I. R. (2012). Overview of random forest methodology and practical guidance with emphasis on computational biology and bioinformatics. Wiley Interdiscip. Rev. Data Min. Knowled. Discov. 2, 493–507. 10.1002/widm.1072

[B8] Bowman N. E. Kording K. P. Gottfried J. A. (2012). Temporal integration of olfactory perceptual evidence in human orbitofrontal cortex. Neuron 75, 916–927. 10.1016/j.neuron.2012.06.035 22958830 PMC3441053

[B9] Breiman L. (2001). Random forests. Mach. Learn. 45, 5–32. 10.1023/A:1010933404324

[B10] Carter R. M. Bowling D. L. Reeck C. Huettel S. A. (2012). A distinct role of the temporal-parietal junction in predicting socially guided decisions. Science 337, 109–111. 10.1126/science.1219681 22767930 PMC3563331

[B11] Chang L. J. Sanfey A. G. (2013). Great expectations: neural computations underlying the use of social norms in decision-making. Soc. Cogn. Affect. Neur. 8, 277–284. 10.1093/scan/nsr094 22198968 PMC3594719

[B12] Collins A. G. Frank M. J. (2014). Opponent actor learning (OpAL): modeling interactive effects of striatal dopamine on reinforcement learning and choice incentive. Psychol. Rev. 121, 337. 10.1037/a0037015 25090423

[B13] De Martino B. Fleming S. M. Garrett N. Dolan R. J. (2013). Confidence in value-based choice. Nature Neuroscience 16(1), 105–110. 10.1038/nn.3279 23222911 PMC3786394

[B14] Dehaene S. Spelke E. Pinel P. Stanescu R. Tsivkin S. (1999). Sources of mathematical thinking: behavioral and brain-imaging evidence. Science 284, 970–974. 10.1126/science.284.5416.970 10320379

[B15] Desikan R. S. Ségonne F. Fischl B. Quinn B. T. Dickerson B. C. Blacker D.. (2006). An automated labeling system for subdividing the human cerebral cortex on MRI scans into gyral based regions of interest. Neuroimage 31, 968–980. 10.1016/j.neuroimage.2006.01.021 16530430

[B16] Destrieux C. Fischl B. Dale A. Halgren E. (2010). Automatic parcellation of human cortical gyri and sulci using standard anatomical nomenclature. Neuroimage 53, 1–15. 10.1016/j.neuroimage.2010.06.010 20547229 PMC2937159

[B17] Dormann C. F. Elith J. Bacher S. Buchmann C. Carl G. Carré G.. (2013). Collinearity: a review of methods to deal with it and a simulation study evaluating their performance. Ecography 36, 27–46. 10.1111/j.1600-0587.2012.07348.x

[B18] Elliott M. L. Knodt A. R. Ireland D. Morris M. L. Poulton R. Ramrakha S.. (2020). What is the test-retest reliability of common task-functional MRI measures? New empirical evidence and a meta-analysis. Psychol. Sci. 31, 792–806. 10.1177/0956797620916786 32489141 PMC7370246

[B19] Esteban O. Blair R. Markiewicz C. J. Berleant S. L. Moodie C. Ma F.. (2018a). “FMRIPrep.” Software. *Zenodo*. 10.5281/zenodo.852659

[B20] Esteban O. Markiewicz C. Blair R. W. Moodie C. Isik A. I. Erramuzpe A.. (2018b). fMRIPrep: a robust preprocessing pipeline for functional MRI. Nat. Methods 16, 111–116. 10.1038/s41592-018-0235-4 30532080 PMC6319393

[B21] Filimon F. Philiastides M. G. Nelson J. D. Kloosterman N. A. Heekeren H. R. (2013). How embodied is perceptual decision making? Evidence for separate processing of perceptual and motor decisions. J. Neurosci. 33, 2121–2136. 10.1523/JNEUROSCI.2334-12.2013 23365248 PMC6619122

[B22] Fischl B. Salat D. H. Busa E. Albert M. Dieterich M. Haselgrove C.. (2002). Whole brain segmentation: automated labeling of neuroanatomical structures in the human brain. Neuron 33, 341–355. 10.1016/S0896-6273(02)00569-X 11832223

[B23] Fyhn M. Molden S. Witter M. P. Moser E. I. Moser M.-B. (2004). Spatial representation in the entorhinal cortex. Science 305, 1258–1264. 10.1126/science.1099901 15333832

[B24] Genon S. Wensing T. Reid A. Hoffstaedter F. Caspers S. Grefkes C.. (2017). Searching for behavior relating to grey matter volume in a-priori defined right dorsal premotor regions: lessons learned. Neuroimage 157, 144–156. 10.1016/j.neuroimage.2017.05.053 28552730 PMC8011585

[B25] Germann J. Petrides M. (2020). Area 8A within the posterior middle frontal gyrus underlies cognitive selection between competing visual targets. Eneuro 7:ENEURO.0102-20.2020. 10.1523/ENEURO.0102-20.2020 32817199 PMC7540933

[B26] Glasser M. F. Coalson T. S. Robinson E. C. Hacker C. D. Harwell J. Yacoub E.. (2016). A multi-modal parcellation of human cerebral cortex. Nature 536, 171–178. 10.1038/nature18933 27437579 PMC4990127

[B27] Gorgolewski K. Burns C. D. Madison C. Clark D. Halchenko Y. O. Waskom M. L.. (2011). Nipype: a flexible, lightweight and extensible neuroimaging data processing framework in Python. Front. Neuroinform. 5:13. 10.3389/fninf.2011.00013 21897815 PMC3159964

[B28] Gorgolewski K. J. Esteban O. Markiewicz C. J. Ziegler E. Ellis D. G. Notter M. P.. (2018). “Nipype.” Software. *Zenodo*. 10.5281/zenodo.596855

[B29] Gradassi A. Slagter S. K. Pinho A. D. S. Molleman L. van den Bos W. (2023). Network distance and centrality shape social learning in the classroom. School Psychol. 38, 67. 10.1037/spq0000490 35511533

[B30] Hampton A. N. Bossaerts P. O'Doherty J. P. (2008). Neural correlates of mentalizing-related computations during strategic interactions in humans. Proc. Natl. Acad. Sci. U S A. 105, 6741–6746. 10.1073/pnas.0711099105 18427116 PMC2373314

[B31] Hofmans L. van den Bos W. (2022). Social learning across adolescence: a Bayesian neurocognitive perspective. Dev. Cognit. Neurosci. 58, 101151. 10.1016/j.dcn.2022.101151 36183664 PMC9526184

[B32] Iscan Z. Jin T. B. Kendrick A. Szeglin B. Lu H. Trivedi M.. (2015). Test–retest reliability of freesurfer measurements within and between sites: effects of visual approval process. Hum. Brain Mapp. 36, 3472–3485. 10.1002/hbm.22856 26033168 PMC4545736

[B33] Keuken M. C. Hardie A. Dorn B. Dev S. Paulus M. Jonas K.. (2011). The role of the left inferior frontal gyrus in social perception: an rTMS study. Brain Res. 1383, 196–205. 10.1016/j.brainres.2011.01.073 21281612

[B34] Kircher T. T. Senior C. Phillips M. L. Benson P. J. Bullmore E. T. Brammer M.. (2000). Towards a functional neuroanatomy of self processing: effects of faces and words. Cognit. Brain Res. 10, 133–144. 10.1016/S0926-6410(00)00036-7 10978701

[B35] Krawczyk D. C. (2002). Contributions of the prefrontal cortex to the neural basis of human decision making. Neurosci. Biobehav. Rev. 26, 631–664. 10.1016/S0149-7634(02)00021-0 12479840

[B36] Lebreton M. Abitbol R. Daunizeau J. Pessiglione M. (2015). Automatic integration of confidence in the brain valuation signal. Nat. Neurosci. 18, 1159–1167. 10.1038/nn.4064 26192748

[B37] Leung C. Cao F. Nguyen R. Joshi K. Aqrabawi A. J. Xia S.. (2018). Activation of entorhinal cortical projections to the dentate gyrus underlies social memory retrieval. Cell Rep. 23, 2379–2391. 10.1016/j.celrep.2018.04.073 29791849

[B38] Llera A. Wolfers T. Mulders P. Beckmann C. F. (2019). Inter-individual differences in human brain structure and morphology link to variation in demographics and behavior. Elife 8:e44443. 10.7554/eLife.44443.025 31268418 PMC6663467

[B39] Lopez-Rojas J. de Solis C. A. Leroy F. Kandel E. R. Siegelbaum S. A. (2022). A direct lateral entorhinal cortex to hippocampal CA2 circuit conveys social information required for social memory. Neuron 110, 1559–1572. 10.1016/j.neuron.2022.01.028 35180391 PMC9081137

[B40] Mahmoodi A. Nili H. Harbison C. Hamilton S. Trudel N. Bang D.. (2023). Causal role of a neural system for separating and selecting multidimensional social cognitive information. Neuron 111, 1152–1164.e6. 10.1016/j.neuron.2022.12.030 36681075 PMC10914676

[B41] McElreath R. Lubell M. Richerson P. J. Waring T. M. Baum W. Edsten E.. (2005). Applying evolutionary models to the laboratory study of social learning. Evol. Hum. Behav. 26, 483–508. 10.1016/j.evolhumbehav.2005.04.003

[B42] Molleman L. Kurvers R. van den Bos W. (2019). Unleashing the BEAST: a brief measure of human social information use. Evolut. Human Behav. 40, 492–499. 10.1016/j.evolhumbehav.2019.06.005

[B43] Molleman L. Van den Berg P. Weissing F. (2014). Consistent individual differences in human social learning strategies. Nat. Commun. 5:3570. 10.1038/ncomms4570 24705692

[B44] Morgan T. J. Rendell L. E. Ehn M. Hoppitt W. Laland K. N. (2012). The evolutionary basis of human social learning. Proc. R. Soc. B Biol. Sci. 279, 653–662. 10.1098/rspb.2011.1172 21795267 PMC3248730

[B45] Moussaïd M. Herzog S. M. Kämmer J. E. Hertwig R. (2017). Reach and speed of judgment propagation in the laboratory. Proc. Nat. Acad. Sci. 114, 4117–4122. 10.1073/pnas.1611998114 28373540 PMC5402452

[B46] Nee D. E. D'Esposito M. (2016). The hierarchical organization of the lateral prefrontal cortex. Elife 5:e12112. 10.7554/eLife.12112.032 26999822 PMC4811776

[B47] Nogueira R. Abolafia J. M. Drugowitsch J. Balaguer-Ballester E. Sanchez-Vives M. V. Moreno-Bote R. (2017). Lateral orbitofrontal cortex anticipates choices and integrates prior with current information. Nat. Commun. 8:14823 10.1038/ncomms14823 28337990 PMC5376669

[B48] Olsson A. Knapska E. Lindström B. (2020). The neural and computational systems of social learning. Nat. Rev. Neurosci. 21, 197–212. 10.1038/s41583-020-0276-4 32221497

[B49] Pedersen M. L. Endestad T. Biele G. (2015). Evidence accumulation and choice maintenance are dissociated in human perceptual decision making. PLoS ONE 10, 1–20. 10.1371/journal.pone.0140361 26510176 PMC4624809

[B50] Pedregosa F. Varoquaux G. Gramfort A. Michel V. Thirion B. Grisel O.. (2011). Scikit-learn: machine learning in Python. J. Mach. Learn. Res. 12, 2825–2830.

[B51] Poldrack R. A. Farah M. J. (2015). Progress and challenges in probing the human brain. Nature 526, 371–379. 10.1038/nature15692 26469048

[B52] Reckless G. E. Ousdal O. T. Server A. Walter H. Andreassen O. A. Jensen J. (2014). The left inferior frontal gyrus is involved in adjusting response bias during a perceptual decision-making task. Brain Behav. 4, 398–407. 10.1002/brb3.223 24944869 PMC4055190

[B53] Rushworth M. F. S. Mars R. B. Sallet J. (2013). Are there specialized circuits for social cognition and are they unique to humans? Curr. Opin. Neurobiol. 23, 436–442. 10.1016/j.conb.2012.11.013 23290767

[B54] Saxe R. Kanwisher N. (2003). People thinking about thinking people: the role of the temporo-parietal junction in “theory of mind.” Neuroimage 19, 1835–1842. 10.1016/S1053-8119(03)00230-1 12948738

[B55] Shipp S. (2017). The functional logic of corticostriatal connections. Brain Struc. Funct. 222, 669–706. 10.1007/s00429-016-1250-9 27412682 PMC5334428

[B56] Strobl C. Boulesteix A.-L. Zeileis A. Hothorn T. (2007). Bias in random forest variable importance measures: illustrations, sources and a solution. BMC Bioinform. 8, 1–21. 10.1186/1471-2105-8-25 17254353 PMC1796903

[B57] Toelch U. Bach D. R. Dolan R. J. (2014a). The neural underpinnings of an optimal exploitation of social information under uncertainty. Soc. Cogn. Affect. Neurosci. 9, 1746–1753. 10.1093/scan/nst173 24194580 PMC4221218

[B58] Toelch U. Bruce M. J. Newson L. Richerson P. J. Reader S. M. (2014b). Individual consistency and flexibility in human social information use. Proc. Royal Soc. 281:20132864. 10.1098/rspb.2013.2864 24352950 PMC3871325

[B59] Toelch U. van Delft M. J. Bruce M. J. Donders R. Meeus M. T. Reader S. M. (2009). Decreased environmental variability induces a bias for social information use in humans. Evol. Hum. Behav. 30, 32–40. 10.1016/j.evolhumbehav.2008.07.00327409075

[B60] Tomasi D. Chang L. Caparelli E. Ernst T. (2007). Different activation patterns for working memory load and visual attention load. Brain Res. 1132, 158–165. 10.1016/j.brainres.2006.11.030 17169343 PMC1831676

[B61] Wang K. Jiang T. Yu C. Tian L. Li J. Liu Y.. (2008). Spontaneous activity associated with primary visual cortex: a resting-state FMRI study. Cereb. Cortex 18, 697–704. 10.1093/cercor/bhm105 17602140

[B62] Yuan X. Ni L. Li H. Zhang D. Zhou K. (2023). The neural correlates of individual differences in numerosity perception: a voxel-based morphometry study. iScience 26:107392. 10.1016/j.isci.2023.107392 37554464 PMC10405316

[B63] Zhang L. Gläscher J. (2020). A brain network supporting social influences in human decision-making. Sci. Adv. 6:eabb4159. 10.1126/sciadv.abb4159 32875112 PMC7438106

