# Supplementary material for: Brain structure correlates of social information use: an exploratory machine learning approach
Source: Front Hum Neurosci. 2024 Jul 2;18:1383630. doi: 10.3389/fnhum.2024.1383630 (PMC11250561; doi:10.3389/fnhum.2024.1383630)
Supplement: Supplementary file 1 [file Data_Sheet_1.docx]

**SUPPLEMENTARY**

**SUPPLEMENTAL METHODS**

**MRI data preprocessing.** The T1-weighted (T1w) image was corrected for intensity non-uniformity (INU) with N4BiasFieldCorrection (Tustison et al., 2010), distributed with ANTs 2.2.0 (Avants, Epstein, Grossman, & Gee, 2008)(RRID:SCR_004757), and used as T1w-reference throughout the workflow. The T1w-reference was then skull-stripped with a Nipype implementation of the antsBrainExtraction.sh workflow (from ANTs), using OASIS30ANTs as target template. Brain tissue segmentation of cerebrospinal fluid (CSF), white-matter (WM) and gray-matter (GM) was performed on the brain-extracted T1w using fast (FSL 5.0.9, RRID:SCR_002823) (Zhang, Brady, & Smith, 2001). Brain surfaces were reconstructed using recon-all (FreeSurfer 6.0.1, RRID:SCR_001847) (Dale, Fischl, & Sereno, 1999), and the brain mask estimated previously was refined with a custom variation of the method to reconcile ANTs-derived and FreeSurfer-derived segmentations of the cortical grey-matter of Mindboggle (RRID:SCR_002438) (Klein et al., 2017). Volume-based spatial normalization to one standard space (MNI152NLin2009cAsym) was performed through nonlinear registration with antsRegistration (ANTs 2.2.0), using brain-extracted versions of both T1w reference and the T1w template. The following template was selected for spatial normalization: ICBM 152 Nonlinear Asymmetrical template version 2009c (Fonov, Evans, McKinstry, Almli, & Collins, 2009) (RRID:SCR_008796; TemplateFlow ID: MNI152NLin2009cAsym]. Many internal operations of *fMRIPrep* use *Nilearn* 0.6.0 ((Abraham et al., 2014), RRID:SCR_001362), mostly within the functional processing workflow. For more details of the pipeline, see the section corresponding workflows in *fMRIPrep*’s documentation.

**SUPPLEMENTAL RESULTS**

**Lasso regression.** Descriptive statistics for the coefficients resulting from the lasso regression including all predictive features can be found in Table S1. The descriptive statistics of the winning model (that contains only predictive features that had non-zero mean coefficients in the first model) can be found in Table S2. Both tables follow the same order of the features.

| **Feature** | **mean*** | **SD*** | **median*** | **95%*** | **min*** | **max*** |
| --- | --- | --- | --- | --- | --- | --- |
| lh_parstriangularis_volume | 3.1709 | 2.9611 | 4.0167 | [0.0000, 8.2688] | 0.0000 | 8.2688 |
| lh_caudalmiddlefrontal_volume | -0.8998 | 1.2065 | -0.3197 | [-4.7148, 0.0000] | -4.7148 | 0.0000 |
| sex_F | 0.7799 | 0.6981 | 1.0778 | [0.0000, 2.0817] | 0.0000 | 2.0817 |
| rh_postcentral_volume | -0.7453 | 0.7682 | -0.7851 | [-2.7595, 0.0000] | -2.7595 | 0.0000 |
| rh_entorhinal_volume | 0.5527 | 0.9998 | 0.0000 | [0.0000, 3.1202] | 0.0000 | 3.1202 |
| Left-Accumbens-area | 0.3997 | 0.7376 | 0.0000 | [0.0000, 2.5570] | 0.0000 | 2.5570 |
| rh_transversetemporal_volume | -0.2755 | 0.5358 | 0.0000 | [-2.5287, 0.0000] | -2.5287 | 0.0000 |
| lh_postcentral_volume | -0.1555 | 0.3418 | 0.0000 | [-2.2575, 0.0000] | -2.2575 | 0.0000 |
| RANDOM | -0.1128 | 0.8012 | 0.0000 | [-1.9322, 0.0000] | -3.6730 | 3.0516 |
| rh_rostralanteriorcingulate_volume | 0.0153 | 0.1133 | 0.0000 | [0.0000, 1.0723] | 0.0000 | 1.0723 |
| lh_entorhinal_volume | 0.0096 | 0.1131 | 0.0000 | [0.0000, 1.3480] | 0.0000 | 1.3480 |
| lh_superiortemporal_volume | -0.0072 | 0.0673 | 0.0000 | [-0.7620, 0.0000] | -0.7620 | 0.0000 |
| lh_parsorbitalis_volume | 0.0056 | 0.0660 | 0.0000 | [0.0000, 0.7861] | 0.0000 | 0.7861 |
| lh_superiorparietal_volume | 0.0008 | 0.0100 | 0.0000 | [0.0000, 0.1186] | 0.0000 | 0.1186 |
| rh_frontalpole_volume | 0.0005 | 0.0056 | 0.0000 | [0.0000, 0.0673] | 0.0000 | 0.0673 |
| lh_fusiform_volume | -0.0003 | 0.0031 | 0.0000 | [-0.0372, 0.0000] | -0.0372 | 0.0000 |
| **Table S1. Lasso regression coefficients.** The descriptive statistics of the coefficients from the loocv of the lasso regression model, including all features. *10^-2^ | | | | | | |

| **Feature** | **mean*** | **SD*** | **median*** | **95%*** | **min*** | **max*** |
| --- | --- | --- | --- | --- | --- | --- |
| lh_parstriangularis_volume | 11.0869 | ±2.8002 | 11.7286 | [0.0000, 14.3919] | 0.0000 | 14.3919 |
| lh_caudalmiddlefrontal_volume | -6.7169 | ±1.8099 | -7.1624 | [-9.5289, 0.0000] | -9.5289 | 0.0000 |
| sex_F | 1.5469 | ±0.4298 | 1.6227 | [0.0000, 2.2153] | 0.0000 | 2.2153 |
| rh_postcentral_volume | -3.7546 | ±1.0872 | -4.0287 | [-6.0427, 0.0000] | -6.0427 | 0.0000 |
| rh_entorhinal_volume | 7.1999 | ±1.9582 | 7.7094 | [0.0000, 9.8249] | 0.0000 | 9.8249 |
| Left-Accumbens-area | 4.5687 | ±1.2969 | 4.8162 | [0.0000, 8.1335] | 0.0000 | 8.1335 |
| rh_transversetemporal_volume | -2.7215 | ±0.8500 | -2.8921 | [-4.8562, 0.0000] | -4.8562 | 0.0000 |
| lh_postcentral_volume | -1.2133 | ±0.5967 | -1.2455 | [-2.9937, 0.0000] | -2.9937 | 0.0000 |
| RANDOM | -0.0596 | ±2.1616 | 0.0000 | [-3.8476, 3.6280] | -7.6086 | 5.4903 |
| rh_rostralanteriorcingulate_volume | 5.5426 | ±1.5905 | 5.9443 | [0.0000, 7.6575] | 0.0000 | 7.6575 |
| lh_entorhinal_volume | 0.1170 | ±0.4468 | 0.0000 | [0.0000, 3.0178] | 0.0000 | 3.0178 |
| lh_superiortemporal_volume | -4.8449 | ±1.4557 | -5.2011 | [-7.4322, 0.0000] | -7.4322 | 0.0000 |
| lh_parsorbitalis_volume | 1.8210 | ±0.7361 | 1.9641 | [0.0000, 3.9206] | 0.0000 | 3.9206 |
| lh_superiorparietal_volume | 2.5509 | ±0.8515 | 2.7033 | [0.0000, 4.3218] | 0.0000 | 4.3218 |
| rh_frontalpole_volume | 3.4961 | ±1.1719 | 3.7069 | [0.0000, 6.6575] | 0.0000 | 6.6575 |
| lh_fusiform_volume | -4.0791 | ±1.2728 | -4.3326 | [-6.7661, 0.0000] | -6.7661 | 0.0000 |
| **Table S2. Lasso regression coefficients winning model.** The descriptive statistics of the coefficients from the loocv of the winning lasso regression model, including only features with a non-zero mean coefficient in the first lasso regression model. *10­^-2^ | | | | | | |


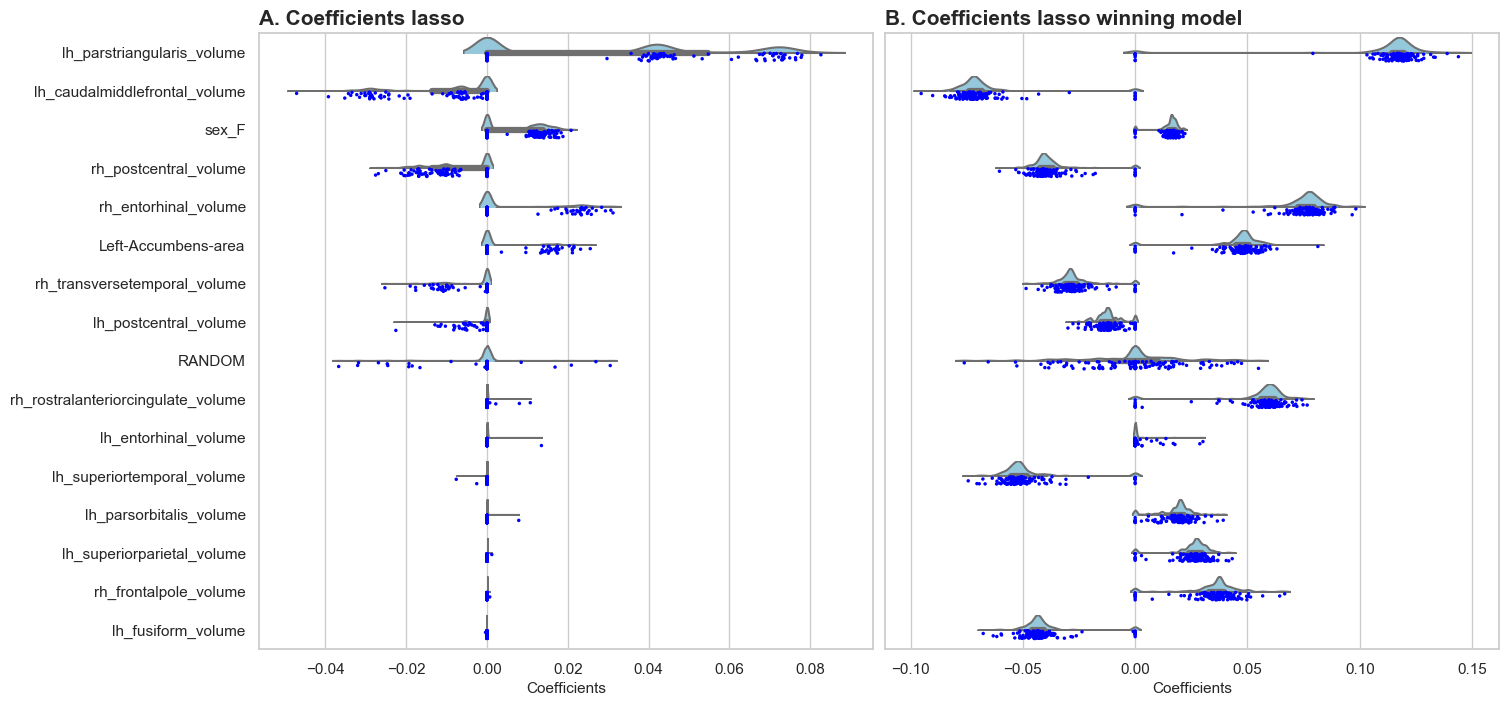


**Figure S1. Lasso regression model coefficients.** A) Distributions of the coefficients resulting from the loocv are shown for each feature that had a non-zero mean coefficient. There are peaks at zero, showing that many single loocv runs resulted in a coefficient of 0 for these features. B) The distributions of the coefficients of the winning model, containing only the non-zero features from the first model. The zero-peaks are much smaller because there is less noise from unimportant features and collinearities. The dots represent the coefficients based on the individual loocv runs.

**Random forest regression.** Descriptive statistics for the permutation importance resulting from the random forest regression including all predictive features can be found in Table S3. The descriptive statistics of the winning model (that contains only predictive features that had a higher mean permutation importance than the baseline feature (RANDOM) in the first model) can be found in Table S4. Both tables follow the same order of the features.

| **Feature** | **mean*** | **SD*** | **median*** | **95%*** | **min*** | **max*** |
| --- | --- | --- | --- | --- | --- | --- |
| lh_parstriangularis_volume | 0.1576 | 0.0187 | 0.1546 | [0.1328, 0.2093] | 0.1174 | 0.2093 |
| lh_postcentral_volume | 0.0857 | 0.0100 | 0.0865 | [0.0722, 0.1165] | 0.0385 | 0.1165 |
| Left-Pallidum | 0.0582 | 0.0078 | 0.0578 | [0.0479, 0.0895] | 0.0408 | 0.0895 |
| rh_paracentral_volume | 0.0376 | 0.0053 | 0.0377 | [0.0286, 0.0555] | 0.0237 | 0.0555 |
| rh_postcentral_volume | 0.0375 | 0.0058 | 0.0383 | [0.0267, 0.0550] | 0.0223 | 0.0550 |
| lh_superiortemporal_volume | 0.0311 | 0.0040 | 0.0310 | [0.0250, 0.0404] | 0.0186 | 0.0404 |
| lh_parsorbitalis_volume | 0.0291 | 0.0051 | 0.0288 | [0.0215, 0.0434] | 0.0160 | 0.0434 |
| Putamen | 0.0251 | 0.0041 | 0.0244 | [0.0198, 0.0379] | 0.0133 | 0.0379 |
| lh_caudalmiddlefrontal_volume | 0.0248 | 0.0036 | 0.0244 | [0.0200, 0.0334] | 0.0159 | 0.0334 |
| Right-Amygdala | 0.0240 | 0.0031 | 0.0241 | [0.0192, 0.0378] | 0.0163 | 0.0378 |
| lh_entorhinal_volume | 0.0225 | 0.0028 | 0.0223 | [0.0188, 0.0330] | 0.0111 | 0.0330 |
| lh_fusiform_volume | 0.0196 | 0.0021 | 0.0194 | [0.0167, 0.0283] | 0.0138 | 0.0283 |
| Right-Pallidum | 0.0195 | 0.0025 | 0.0195 | [0.0164, 0.0279] | 0.0069 | 0.0279 |
| -VentralDC | 0.0183 | 0.0035 | 0.0178 | [0.0141, 0.0332] | 0.0101 | 0.0332 |
| rh_supramarginal_volume | 0.0182 | 0.0026 | 0.0179 | [0.0143, 0.0280] | 0.0090 | 0.0280 |
| rh_parsopercularis_volume | 0.0182 | 0.0020 | 0.0182 | [0.0148, 0.0245] | 0.0116 | 0.0245 |
| Left-Accumbens-area | 0.0175 | 0.0030 | 0.0173 | [0.0133, 0.0266] | 0.0083 | 0.0266 |
| CC_Posterior | 0.0168 | 0.0019 | 0.0169 | [0.0137, 0.0222] | 0.0109 | 0.0222 |
| rh_lateralorbitofrontal_volume | 0.0163 | 0.0027 | 0.0159 | [0.0126, 0.0272] | 0.0107 | 0.0272 |
| lh_lateralorbitofrontal_volume | 0.0162 | 0.0025 | 0.0158 | [0.0127, 0.0235] | 0.0104 | 0.0235 |
| lh_inferiortemporal_volume | 0.0156 | 0.0031 | 0.0148 | [0.0115, 0.0254] | 0.0094 | 0.0254 |
| lh_insula_volume | 0.0154 | 0.0020 | 0.0151 | [0.0128, 0.0202] | 0.0104 | 0.0202 |
| rh_temporalpole_volume | 0.0151 | 0.0021 | 0.0151 | [0.0113, 0.0197] | 0.0093 | 0.0197 |
| rh_superiorfrontal_volume | 0.0151 | 0.0020 | 0.0150 | [0.0121, 0.0226] | 0.0113 | 0.0226 |
| RANDOM | 0.0151 | 0.0115 | 0.0109 | [0.0048, 0.0369] | 0.0048 | 0.0829 |
| **Table S3. Random Forest regression** **permutation importance.** The descriptive statistics of the permutation importances from the loocv of the random forest regression model. *****10^-2^ | | | | | | |

| **Feature** | **mean*** | **SD*** | **median*** | **95%*** | **min*** | **max*** |
| --- | --- | --- | --- | --- | --- | --- |
| lh_parstriangularis_volume | 0.2010 | ±0.0198 | 0.2005 | [0.1702, 0.2534] | 0.1517 | 0.2534 |
| lh_postcentral_volume | 0.1387 | ±0.0137 | 0.1412 | [0.1159, 0.1666] | 0.0797 | 0.1666 |
| Left-Pallidum | 0.0990 | ±0.0109 | 0.0987 | [0.0828, 0.1379] | 0.0709 | 0.1379 |
| rh_paracentral_volume | 0.0846 | ±0.0089 | 0.0858 | [0.0700, 0.1049] | 0.0634 | 0.1049 |
| rh_postcentral_volume | 0.0738 | ±0.0087 | 0.0747 | [0.0594, 0.1051] | 0.0527 | 0.1051 |
| lh_superiortemporal_volume | 0.0636 | ±0.0061 | 0.0639 | [0.0524, 0.0807] | 0.0410 | 0.0807 |
| lh_parsorbitalis_volume | 0.0528 | ±0.0068 | 0.0529 | [0.0424, 0.0729] | 0.0324 | 0.0729 |
| Putamen | 0.0445 | ±0.0050 | 0.0440 | [0.0377, 0.0596] | 0.0313 | 0.0596 |
| lh_caudalmiddlefrontal_volume | 0.0555 | ±0.0060 | 0.0552 | [0.0465, 0.0695] | 0.0432 | 0.0695 |
| Right-Amygdala | 0.0561 | ±0.0045 | 0.0564 | [0.0486, 0.0667] | 0.0430 | 0.0667 |
| lh_entorhinal_volume | 0.0509 | ±0.0041 | 0.0509 | [0.0453, 0.0640] | 0.0322 | 0.0640 |
| lh_fusiform_volume | 0.0421 | ±0.0036 | 0.0424 | [0.0363, 0.0523] | 0.0289 | 0.0523 |
| Right-Pallidum | 0.0441 | ±0.0040 | 0.0440 | [0.0392, 0.0570] | 0.0225 | 0.0570 |
| -VentralDC | 0.0356 | ±0.0044 | 0.0350 | [0.0292, 0.0553] | 0.0263 | 0.0553 |
| rh_supramarginal_volume | 0.0403 | ±0.0035 | 0.0404 | [0.0344, 0.0498] | 0.0286 | 0.0498 |
| rh_parsopercularis_volume | 0.0404 | ±0.0032 | 0.0406 | [0.0351, 0.0485] | 0.0298 | 0.0485 |
| Left-Accumbens-area | 0.0379 | ±0.0040 | 0.0375 | [0.0320, 0.0502] | 0.0293 | 0.0502 |
| CC_Posterior | 0.0462 | ±0.0035 | 0.0463 | [0.0397, 0.0538] | 0.0341 | 0.0538 |
| rh_lateralorbitofrontal_volume | 0.0346 | ±0.0044 | 0.0336 | [0.0285, 0.0494] | 0.0242 | 0.0494 |
| lh_lateralorbitofrontal_volume | 0.0390 | ±0.0038 | 0.0389 | [0.0331, 0.0507] | 0.0303 | 0.0507 |
| lh_inferiortemporal_volume | 0.0391 | ±0.0053 | 0.0385 | [0.0311, 0.0549] | 0.0266 | 0.0549 |
| lh_insula_volume | 0.0370 | ±0.0035 | 0.0368 | [0.0315, 0.0462] | 0.0304 | 0.0462 |
| rh_temporalpole_volume | 0.0339 | ±0.0026 | 0.0340 | [0.0292, 0.0409] | 0.0260 | 0.0409 |
| rh_superiorfrontal_volume | 0.0401 | ±0.0034 | 0.0401 | [0.0348, 0.0501] | 0.0331 | 0.0501 |
| RANDOM | 0.0435 | ±0.0325 | 0.0314 | [0.0181, 0.0874] | 0.0181 | 0.1969 |
| **Table S4. Random Forest regression permutation importance winning model.** The descriptive statistics of the permutation importances from the loocv of the winning random forest regression model, including only features with a mean permutation importance higher than the baseline feature (RANDOM) in the first random forest model . *****10^-2^ | | | | | | |


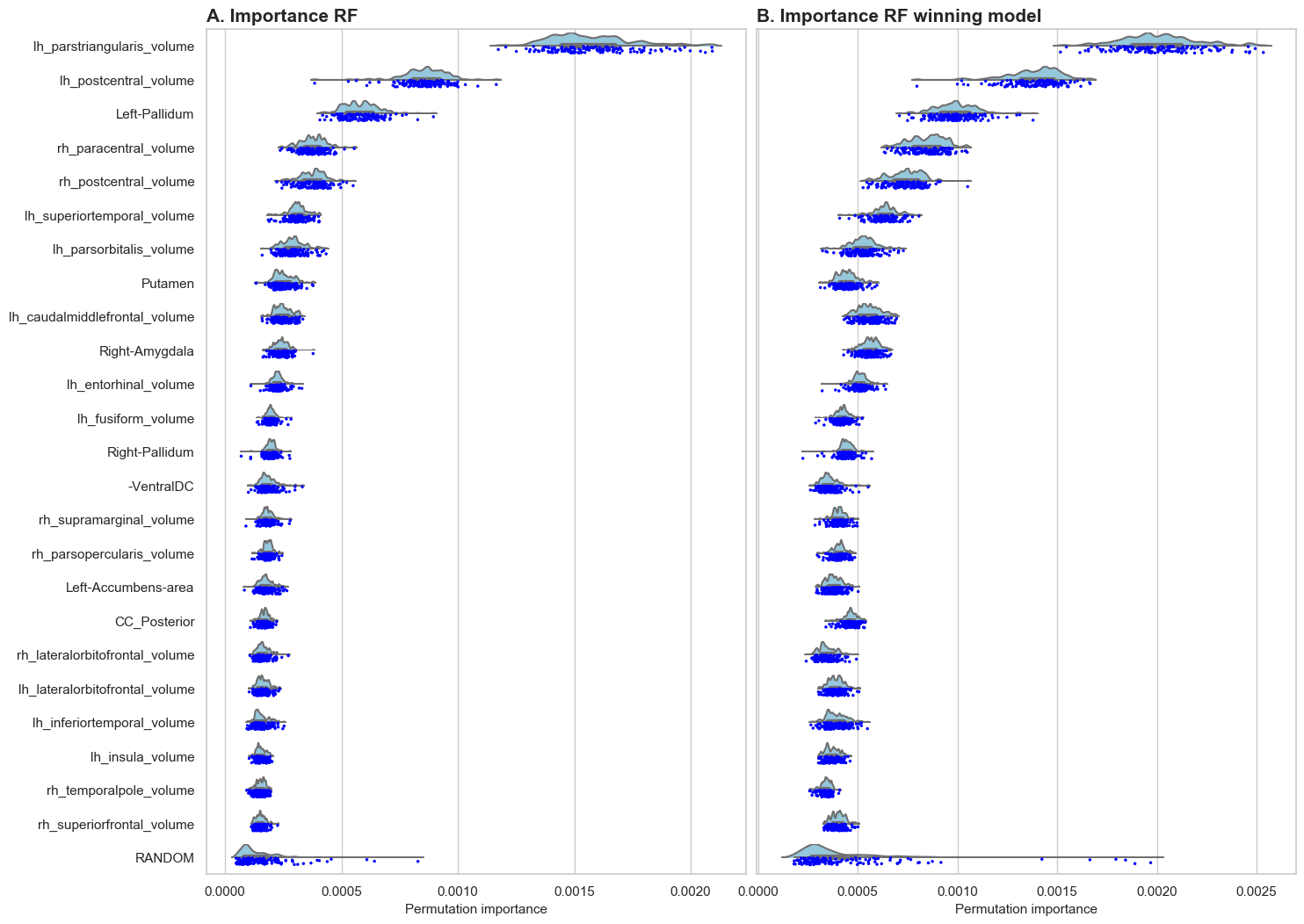
**Figure S2. RF regression model permutation importances.** A) Distributions of the importances resulting from the loocv are shown for each feature that had a higher mean importance than the baseline feature (RANDOM). B) The distributions of the importances of the winning model, containing only the features from the first model that scored higher than the baseline feature. The dots represent the importance based on an individual model.

**Models with S = 0.** To be transparent about possible influences of excluding participants with a mean social information use of zero, the main analysis is run again including these participants. The distribution of the coefficients resulting from the lasso regression including participants with no social information use can be found in Figure S1. The distribution of the permutation importances resulting from the random forest regression including participants with zero social information use can be found in Figure S2. Based on these figures, we can see that the grey matter volume of the left pars triangularis is robustly the biggest predictor for social information use. This is a similar conclusion as from our main analysis. Moreover, we can still see a significant positive correlation for the left pars triangularis with social information use for the dataset including participants with no social information (r = 0.2578, p = 0.001, Table S5). Regarding other features, some slight shifts can be observed in Figure S1, Figure S2, and Table S5.

| **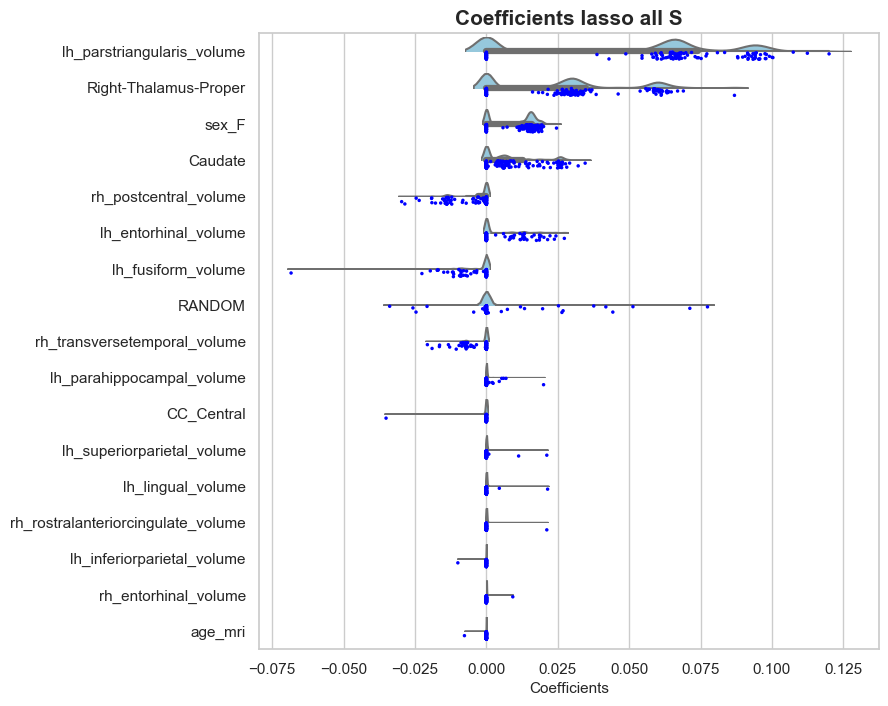** |
| --- |
| **Figure S3. Lasso regression model coefficients including participants with no social information use.** Distributions of the coefficients resulting from the loocv are shown for each feature that had a non-zero mean coefficient. The dots represent the coefficient based on an individual model. |
| **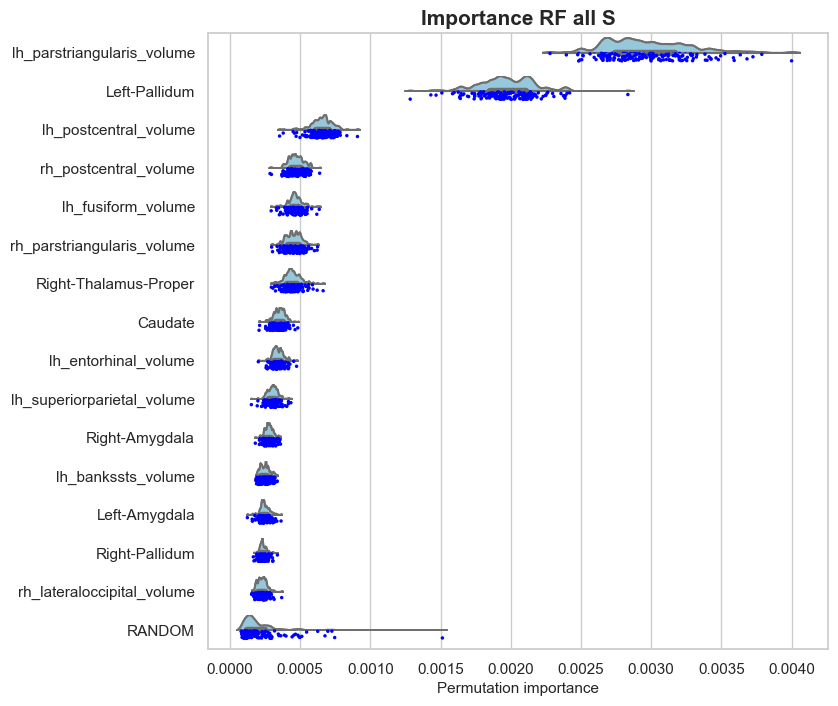** |
| **Figure S4. Random forest regression model permutation importance including participants with no social information use.** Distributions of the permutation importances resulting from the loocv are shown for each feature that had higher mean importance than the baseline feature (RANDOM). The dots represent the importance based on an individual model. |

**Correlations with S = 0**

|  | **S > 0** | | **S ≥ 0** | |
| --- | --- | --- | --- | --- |
|  | **S (p)** | **Abs error (p)** | **S (p)** | **Abs error (p)** |
| lh_parstriangularis_volume | 0.2607  (0.0018) | - | 0.2578 (0.001) | - |
| rh_parstriangularis_volume | - | - | - | - |
| lh_postcentral_volume | -0.1769 (0.0359) | - | - | - |
| rh_postcentral_volume | -0.1803 (0.0324) | -0.2371 (0.0046) | - | -0.2507  (0.0014) |
| lh_caudalmiddlefrontal_volume | -0.1900 (0.0241) | -0.2227 (0.008) | - | -0.2638  (0.0008) |
| rh_caudalmiddlefrontal_volume | - | - | - | - |
| Left-Pallidum | - | - | 0.1804 (0.0229) | - |
| Right-Pallidum | - | - | - | - |
| lh_entorhinal_volume | 0.1729 (0.0404) | - | 0.1683 (0.0339) | - |
| rh_entorhinal_volume | 0.1774 (0.0354) | - | - | - |
| Right-Thalamus-Proper | - | - | 0.2048 (0.0096) | - |
| Left-Thalamus-Proper | - | - | - | - |
| **Table S5.** Correlations with social information use (S) and task performance (Abs error) for the dataset excluding (S > 0) and the dataset including (S ≥ 0) participants with no social information use. P-values of the correlations are shown within parentheses. Only the features with a p-value (in parentheses) lower than 0.05 are shown. | | | | |

**Model performance.** The distribution of the mean squared error (MSE) of the baseline, lasso and random forest model can be found in Figure S3, together with the scores of the winning lasso and random forest model.

| **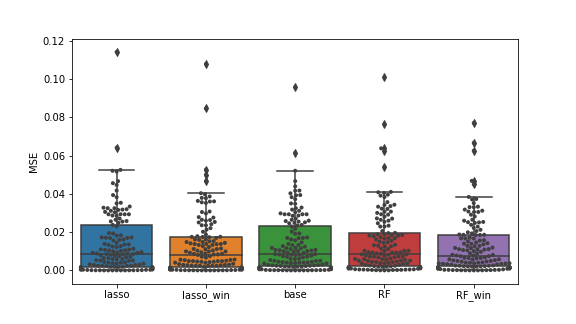** |
| --- |
| **Figure S5. Model performance distribution.** Model performance is calculated as the mean squared error (MSE) for each model from the loocv (n = 141). The baseline model (base) predicts the average social information use of the train data. The random forest models are labelled by RF. The MSE of the winning models slightly decreased (lasso_win and RF_win). |

**Vertex-wise analysis of the relationship between social information use and cortical brain volume**

As an additional exploratory analysis, we assessed the relationship between social information use and cortical brain volume using a vertex-wise approach. After surface reconstruction as described in the methods section, all individual structural maps were concatenated to into a single dataset. We then correlated individual’s social information use with their cortical volume measures, using a FWHM kernel of 10 mm, an initial vertex-wise cluster-forming threshold of *p* = 0.001 and a cluster-wise threshold of *p* = 0.05. Figure S6 shows the areas that survive this threshold, as well as a less stringent threshold. Of note, this vertex-wise approach assesses simple correlations and does not account for any non-linear relationships or partial correlations. It can therefore be considered complementary to our main analyses.


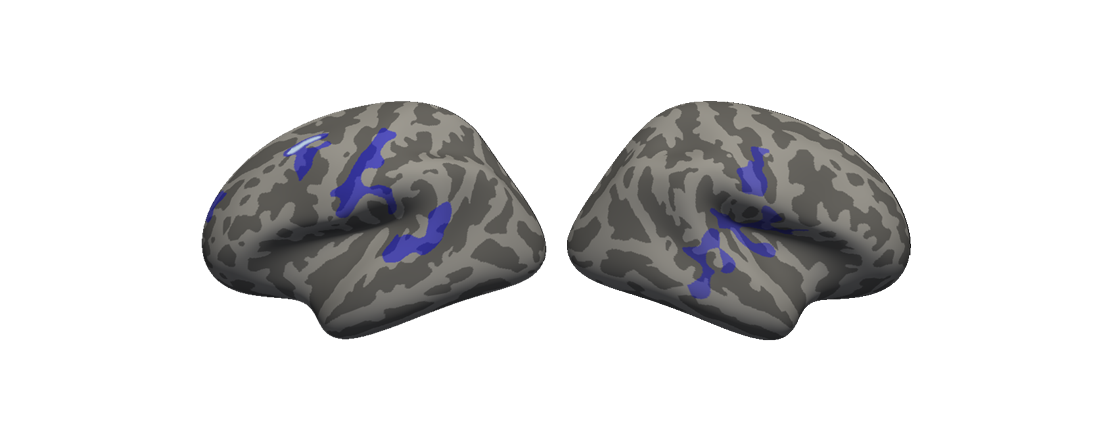


**Figure S6. Vertex-wise analysis of the relationship between social information use and cortical brain volume.** The light blue cluster was significant at an initial vertex-wise cluster-forming threshold of *p* = 0.001 and a cluster-wise threshold of *p* = 0.05: peak t-value = -4.17 (x = -34, y = 4, z = 55; caudal middle frontal gyrus). The more transparent dark blue areas do not survive our threshold, but do survive the less stringent initial cluster-forming threshold of *p* = 0.01 and cluster-wise threshold of *p* = 0.05.

**SUPPLEMENTAL REFERENCES**

Abraham, A., Pedregosa, F., Eickenberg, M., Gervais, P., Mueller, A., Kossaifi, J., . . . Varoquaux, G. (2014). Machine learning for neuroimaging with scikit-learn. *Frontiers in neuroinformatics, 8*, 14. doi:<https://doi.org/10.3389/fninf.2014.00014>

Avants, B. B., Epstein, C. L., Grossman, M., & Gee, J. C. (2008). Symmetric diffeomorphic image registration with cross-correlation: evaluating automated labeling of elderly and neurodegenerative brain. *Medical image analysis, 12*(1), 26-41. doi:<https://doi.org/10.1016/j.media.2007.06.004>

Dale, A. M., Fischl, B., & Sereno, M. I. (1999). Cortical surface-based analysis: I. Segmentation and surface reconstruction. *Neuroimage, 9*(2), 179-194. doi:<https://doi.org/10.1006/nimg.1998.0395>

Fonov, V. S., Evans, A. C., McKinstry, R. C., Almli, C., & Collins, D. (2009). Unbiased nonlinear average age-appropriate brain templates from birth to adulthood. *Neuroimage*(47), S102. doi:<https://doi.org/10.1016/S1053-8119(09)70884-5>

Klein, A., Ghosh, S. S., Bao, F. S., Giard, J., Häme, Y., Stavsky, E., . . . Chaibub Neto, E. (2017). Mindboggling morphometry of human brains. *PLoS computational biology, 13*(2), e1005350. doi:<https://doi.org/10.1371/journal.pcbi.1005350>

Tustison, N. J., Avants, B. B., Cook, P. A., Zheng, Y., Egan, A., Yushkevich, P. A., & Gee, J. C. (2010). N4ITK: improved N3 bias correction. *IEEE transactions on medical imaging, 29*(6), 1310-1320. doi:<https://doi.org/10.1109/TMI.2010.2046908>

Zhang, Y., Brady, M., & Smith, S. (2001). Segmentation of brain MR images through a hidden Markov random field model and the expectation-maximization algorithm. *IEEE Trans Med Imaging, 20*(1), 45-57. doi:<https://doi.org/10.1109/42.906424>
